# Natural processes dominate the pollution levels during COVID-19 lockdown over India

**DOI:** 10.1038/s41598-021-94373-4

**Published:** 2021-07-23

**Authors:** Venkat Ratnam Madineni, Hari Prasad Dasari, Ramakrishna Karumuri, Yesubabu Viswanadhapalli, Prasad Perumal, Ibrahim Hoteit

**Affiliations:** 1 National Atmospheric Research Laboratory, Gadanki, Andhra Pradesh India; 2 Physical Sciences and Engineering Division, King Abdullah University of Science and Technology, Thuwal, Saudi Arabia

**Keywords:** Environmental chemistry, Environmental impact

## Abstract

The lockdown measures that were taken to combat the COVID-19 pandemic minimized anthropogenic activities and created natural laboratory conditions for studying air quality. Both observations and WRF-Chem simulations show a 20–50% reduction (compared to pre-lockdown and same period of previous year) in the concentrations of most aerosols and trace gases over Northwest India, the Indo Gangetic Plain (IGP), and the Northeast Indian regions. It is shown that this was mainly due to a 70–80% increase in the height of the boundary layer and the low emissions during lockdown. However, a 60–70% increase in the pollutants levels was observed over Central and South India including the Arabian sea and Bay of Bengal during this period, which is attributed to natural processes. Elevated (dust) aerosol layers are transported from the Middle East and Africa via long-range transport, and a decrease in the wind speed (20–40%) caused these aerosols to stagnate, enhancing the aerosol levels over Central and Southern India. A 40–60% increase in relative humidity further amplified aerosol concentrations. The results of this study suggest that besides emissions, natural processes including background meteorology and dynamics, play a crucial role in the pollution concentrations over the Indian sub-continent.

## Introduction

The declaration that SARS-CoV-2 (COVID-19) had reached pandemic proportions by the World Health Organization (WHO) in early 2020 led to a global standstill in which several countries enforced a complete or partial lockdown of businesses and movement^1^. As of March 2021, approximately 124 million COVID-19 positive cases have been reported globally, 11 millions of which were in India, the country with the second highest population in the world. Although the virus was discovered before the end of 2019, COVID-19 was labelled as severe over the first few months of 2020, across the world. As no medicines have yet been approved for the treatment of COVID-19 and the global percentage of vaccinated people are low, governments remains continue to implement containment measures (social distancing) to minimize the spread of the virus. Different countries have followed different approaches in terms of lockdown measures, including complete lockdowns, the closure of non-essential services, or shutting specific businesses. As a result, significant improvements in the Air Quality Index (AQI) have been reported over several countries around the globe^2^, including Brazil^3^, China^4–9^, Ecuador^10^, South Korea^11^, western Europe^11^, Iran^12,13^, India^14,15^, Malaysia^16^, Spain^17^, and the USA^11,18^. A reduction of 17% in global CO_2_ compared to 2019 levels was reported by Le Quéré et al.^19^, although Safarian et al.^20^ reported only a 7% reduction. An increase in O_3_ levels because of the reduction in NO_x_ concentrations has also been observed across several countries^5,6,12,16,21,22^, but 10% reduction was estimated over rural location. Very few studies [e.g.,^23,24^] have reported an increase in the concentration of pollutants as a result of prevailing atmospheric conditions, although several studies^25–30^ have stressed the role of meteorological parameters (mostly temperature and humidity) on outbreaks of COVID-19.

The first positive case of COVID-19 in India was reported in the state of Kerala on January 30, 2020, which was followed by several cases throughout February^31^ and a gradual increase in the number of cases during March. The peak that occurred around the third week of March forced the government of India to implement containment measures. This started with a (Bharat/Janatha) curfew on March 22, 2020 followed by a complete lockdown that was carried out over four phases. Public gatherings were banned, shopping malls, cinema halls, and prayer halls were closed, and wedding celebrations were prohibited, with many more restrictions put into place. A strict countrywide first-phase lockdown (lockdown-1) was implemented for 21 days from March 25 to April 14, 2020, which included suspension of all business activities as well as industries, transport (air, water, and road), markets, shops, tourism, construction, and hotels, while retaining essential services. The lockdown was extended until May 03, 2020 (lockdown-2), after which some restrictions on industrial and construction activities were relaxed. The lockdown was then extended until May 17 (lockdown-3), and again until May 31, 2020 (lockdown-4), after which the restrictions on most of the activities were relaxed, except for public transportation and mass gatherings.

National Aeronautics and Space Administration (NASA) satellite images (https://earthsky.org/earth/satellite-images-air-pollution-india-covid19) showed significant improvements in the air quality over India and the surrounding regions during the first lockdown. The restrictive measures taken by the government of India to minimize the spread of COVID-19 improved the air quality standards as a result of the significant reduction in anthropogenic activities. Nitrogen oxides, also known as NO_x_, are primary sources of pollutants generated by vehicles and industry^6^. Other gaseous pollutants such as carbon monoxide (CO), sulfur dioxide (SO_2_), methane (CH_4_), tropospheric ozone (O_3_), PM_2.5_, and PM_10_ are all emitted by anthropogenic activities (power plants, oil refineries, vehicular traffic, mining, etc.). Sharma et al.^32^ compared the concentration of pollutants over 22 Indian cities during the lockdown periods to those during the same period in previous years (2017 to 2019) and reported significant reductions of 43%, 31%, 10%, and 18% in PM_2.5_, PM_10_, CO, and NO_2_ concentrations_,_ respectively. The study also reported almost negligible changes in SO_2_, but an unexpected increase (of 17%) in O_3_ concentrations during the lockdown. A similar analysis performed by Jain and Sharma^15^, in which the concentration of pollutants in five Indian megacities during the period March–April 2020 were compared with those during the same period in 2019, reported significant reductions in the concentrations of PM_2.5_, PM_10_, NO_2_, and CO. An AQI assessment over New Delhi^33^ also indicated a significant reduction in pollution, such as a 50% reduction in coarse and fine particulate matter (PM_10_ and PM_2.5_), a 52% reduction in NO_2_, and a 30% increase in CO concentrations. All these studies suggested however a clear increase in O_3_ during the lockdown, which was attributed to changes in the amount of NO_x_ and volatile organic compounds in the atmosphere. This is because O_3_ is formed in the lower atmosphere via the reaction of NO_x_ with volatile organic compounds in the presence of sunlight^34,35^. An increase in O_3_ was also reported in Rome, Turin, and Wuhan during lockdown, by 14%, 27%, and 36%, respectively^35^.

All the studies referred to above are limited to point measurements and the spatial distribution of few parameters (Aerosol Optical Depth, NO_2_, and SO_2_) obtained from satellite observations. Singh et al.^36^ reported about 30–70% reduction in NO_2_, 40–60% in PM_2.5_ and PM_10_ and 20–40% in CO, subject to large spatial variations, after analyzing data from 134 Central Pollution Control Board (CPCB) stations. However, no attempt has yet been made to understand the underlying physical mechanisms that contribute to the changes in the AQI during lockdown. In this study, a state-of-the-art advanced Weather Research Forecasting (WRF) model coupled with a Chemistry module (WRF-Chem) was used along with satellite observations to investigate the possible physical mechanisms that contributed to the changes in pollution levels over the Indian sub-continent during lockdown.

## Materials and methods

WRF-Chem version 3.9.1^37^ was implemented to simulate the meteorological and atmospheric chemistry conditions over the Indian Sub-continent. Several studies^38–43^ have demonstrated the ability of WRF-Chem to capture the spatio-temporal distribution of aerosols, air quality at the regional scale, and cloud-chemistry interactions by resolving the interactions between aerosols, trace gas reactions, emissions, mixing, transport, deposition, chemical transformations, and photolysis.

In this study, we implemented the WRF-Chem^37^ with 90 vertical levels and a horizontal resolution of 30 km covering both Asia and the regions around the Indian Ocean (Fig. S1). The model initial and boundary conditions were extracted from the Final reanalysis (FNL) data, which are available at a 1° × 1° spatial resolution. The time-varying low boundary conditions of sea surface temperature are taken from the NCEP real-time global high-resolution data. The model was integrated from 00:00 UTC on February 20 until 00:00 UTC on May 01, 2020. The first 15 day of the simulation were treated as spin-up and thus excluded from the analysis. The remaining period over the different phases of lockdown in India was used for the analysis.

We used the EDGAR-HTAP V2.2 anthropogenic emission data in the WRF-Chem simulations during the pre-lockdown period. This emission datasets were generated in 2010 by collecting local information from regional inventories to produce a global inventory of emissions. The resulting emissions are mapped on the model grid using scaling factors suggested for India by Venkataraman et al.^44^ to describe the updated emissions scenarios during the pre-lockdown period. We have further conducted sensitivity experiments with WRF-Chem to estimate the percentage reduction in emissions from different emission sectors over India. The scaling factor was selected after conducting several experiments in which we changed the percentage of reductions in the emissions (between 30 and 70%) based on the recent COVID-19 observational studies^15,32,36,44,45^. From these simulations, we found that an overall emission reduction of 40% in the anthropogenic emission inventory is able to reproduce a realistic estimate of the observed concentrations during the COVID-19 lockdown. We thus utilized this scaling factor of 40% reduction to represent the changes due to the impact of COVID-19 in the anthropogenic emissions inventory. The chemical species included in the anthropogenic emissions consists of CO, SO_2_, NOx, NH_3_, NMVOCs, Black Carbon (BC), organic carbon (OC), PM_2.5_ and PM_10_. Though the configured scaling factor used in this study may not represent the real scenarios of emissions during the COVID-19 lockdown, it still provides a reasonable approximation of the overall reduction associated with the lockdown.

In WRF-Chem, since the emissions inventory acts as a mainly background, the supply of realistic initial and boundary conditions of chemical fields is critical step in determining the accuracy of the modeling system. In this study, we have supplied initial and boundary conditions obtained from the assimilated fields of Whole Atmosphere Community Climate Model (WACCM). This reanalysis product as one standard data used for the initialization of WRF-Chem model as it assimilates all available observations of different chemical species using improved assimilation algorithms^46^. The complete details of the experimental design, model physics, datasets, and measurements used in this study are provided in the Supplementary Material.

A combination of satellite observations and WRF-Chem simulations were used to investigate the changes in the aerosol and trace-gas distribution over India and the adjacent regions during lockdown, from March 8 to April 20, 2020, which includes the first phase of total lockdown that was implemented from March 25 to April 14, 2020. To examine the effect of lockdown on trace gases and aerosols, we separated the total simulation period (March 1 to May 1, 2020) into two sub-periods; pre-lockdown (hereafter referred to as PLD) (March 8 to 21, 2020) and lockdown (hereafter referred to as DLD) (March 25 to April 20, 2020). The percentage change in aerosol and trace gas concentrations between PLD and DLD was estimated as:

1 $$\text{Percentage} \; \text{change}\left({\%}\right)=\frac{\text{DLD}\; \text{concentration}-\text{PLD} \; \text{concentration}}{\text{PLD} \; \text{concentration}}.$$

Similarly, the percentage change in aerosol and trace gas concentrations between 2020 and 2019 was estimated as:

2 $$\text{Percentage} \; \text{change}\left({\%}\right)=\frac{2020 \; \text{ concentration}-2019 \; \text{ concentration}}{2019 \; \text{ concentration}}.$$

The focus of this study is to investigate the effects of lockdown on aerosol and trace gases over India, including the Arabian sea and the Bay of Bengal (BoB). The aerosol and trace gas concentrations do not significantly vary within the boundary layer. To remove the topographic/surface effects, we analyzed the mean concentrations averaged between 1000 and 800 hPa as simulated by WRF-Chem. The study period falls within the pre-Indian summer monsoon season, during which the well-mixed boundary layer often reaches 1.5–2 km^47^. Thus, the integrated mean model values should not affect the observed major features. Furthermore, the prevailing weather conditions play a dominant role in the variations observed in the detected emissions, which may exhibit variability on a seasonal to inter-annual scales. We have therefore provided a detailed analysis of the background meteorological conditions over the study region in the Supplementary Material.

## Results

### Validation of WRF-Chem outputs

**Figure 1:**
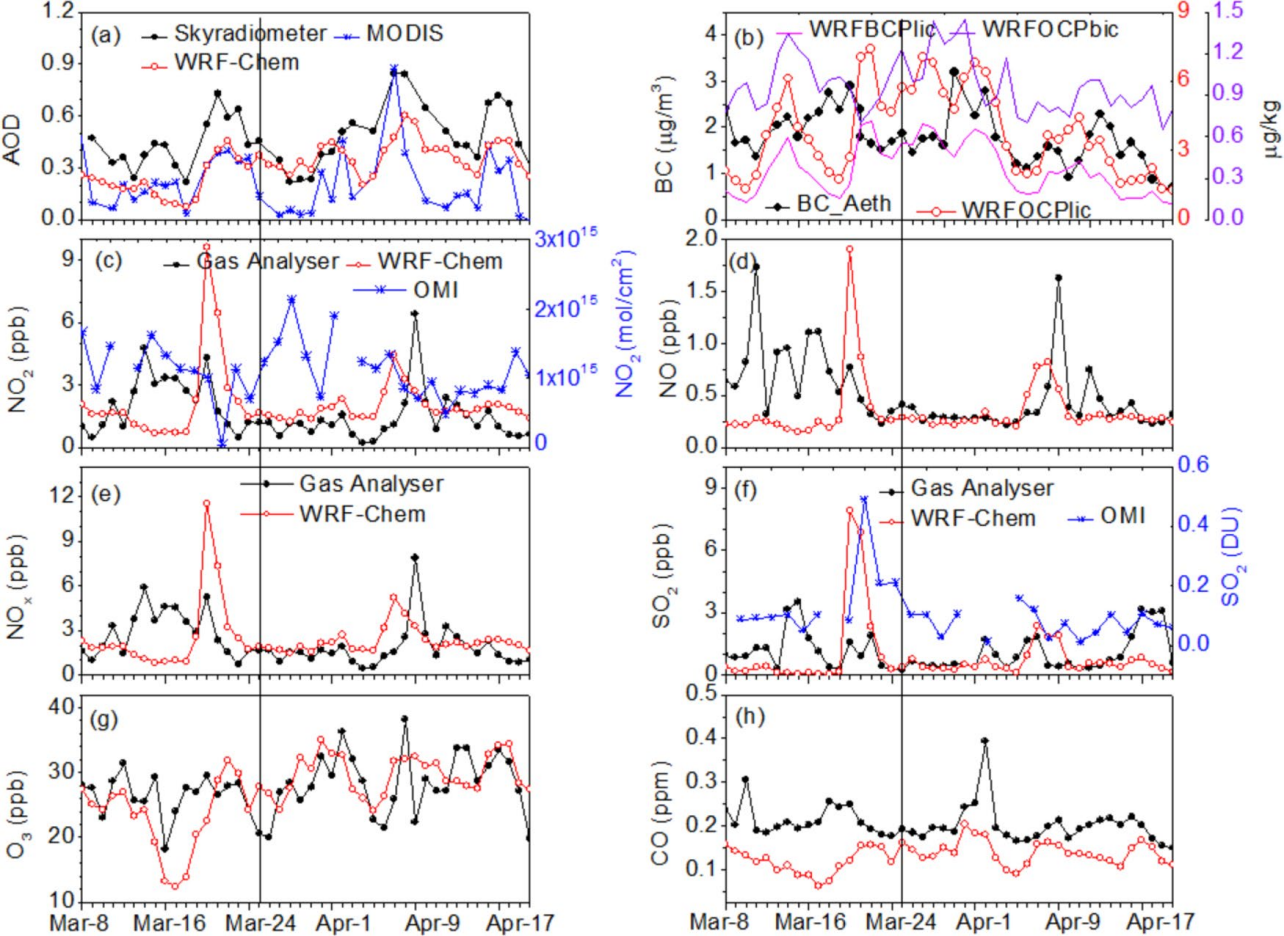
Time series for (**a**) AOD, (**b**) BC, (**c**) NO_2_, (**d**) NO, (**e**) NO_x_, (**f**) SO_2_, (**g**) O_3_, and (**h**) CO obtained from WRF-Chem model simulations for March 8 to April 20, 2020 over the Gadanki region. Trace gases obtained from a gas analyser at Gadanki are superimposed on the respective panels (**c**–**h**). Skyradiometer AOD and Aethalometer BC are also superimposed in (**a**) and (**b**), respectively. Hydrophilic and hydrophobic BC and OC as simulated by WRF-Chem are also shown in (**a**). MODIS AOD is superimposed in (**a**). NO_2_ and SO_2_ from OMI are superimposed in (**c**) and (**f**), respectively. Note that model simulated O_3_ and CO are doubled and halved, respectively. WRF-Chem simulated outputs are represented by red lines, ground-based observations by black lines, and satellite observations by blue lines. The vertical line indicates the date on which the lockdown began. The figures are plotted using ORIGIN software (https://www.originlab.com).

The WRF-Chem outputs were first validated using calibrated ground-based and space-borne measurements. The details of the ground-based measurements, their collective protocols, and accuracy are included in Supplementary Material. Daily mean aerosol (AOD and Black Carbon) and trace gas parameters (NO, NO_2_, NO_x_, SO_2_, O_3_, and CO) obtained for Gadanki (13.5°N, 79.2°E) from surface measurements and WRF-Chem show (Fig. 1) that the model is able to capture the day-to-day variations similar to the ground-based surface observations. The simulated NO_2_, NO, NO_x_, and SO_2_ concentrations and the associated trends demonstrate very good agreement with the surface observations (Fig. 1c–f). However, although the simulated O_3_ and CO reproduced the observed patterns, their concentrations were almost double and half those of the ground-based observations, respectively (Fig. 1g,h). Small peaks in the surface measurements (except O_3_ and CO) that occurred during March 14–20, 2020, were due to local emissions and were not reproduced by WRF-Chem. A slight time lag is observed in the maximum values simulated by WRF-Chem, which may be due to the relatively coarse model grid that does not fully resolve the mesoscale processes^48^. The small differences between the ground-based observations and WRF-Chem simulations can also be attributed to differences in sampling size and local emissions.

The instantaneous measurements of AOD and NO_2_ (SO_2_) from Moderate Resolution Imaging Spectroradiometer (MODIS) and Ozone Monitoring Instrument (OMI) at the closest point (78.5–80.5°E, 12.5–14.5°N) to Gadanki (Fig. 1a,c,f) in the model grid indicates that the model is able to reproduce the observed variations, albeit to a slight overestimation (underestimation) of the AOD compared to MODIS (Sky-radiometer). The AOD reached as high as 0.6 during the PLD period, which was followed by a gradual decrease during the DLD before reaching a minimum of 0.2. Interestingly, all the observations show an increase in the AOD during the first week of April 2020. Although a one-to-one comparison between the observed and the WRF-Chem simulated hydrophilic and hydrophobic Black Carbon (BC) and Organic Carbon (OC) is not possible (as the BC obtained using an Aethalometer cannot be separated), it is clear that the hydrophilic BC and OC match (Fig. 1b) well with the results obtained for BC using the Aethalometer. WRF-Chem outputs slightly overestimates (underestimates) the concentrations of NO_2_ (Fig. 1b) and SO_2_ (Fig. 1f) compared to the ground-based trace gas analyzer (OMI satellite) measurements; however, the day-to-day variations were successfully reproduced. A large increase in all concentrations that were observed during March 13–18, 2020 by ground-based instruments was due to a highly localized event and is therefore not captured by WRF-Chem nor the satellite measurements limited by the sampling issues from polar orbiting platform. Apart from these limitations, the failure of MODIS and OMI to capture the peak values can be related to the polar orbits of these platforms (with only two visits per day). Despite some slight shifting in the peaks, the day-to-day variations match well, particularly during the DLD period. Moreover, a sharp decrease in the concentrations is noticeable and the values almost reach the limits of detection during the DLD (Fig. 1c–h).

**Figure 2:**
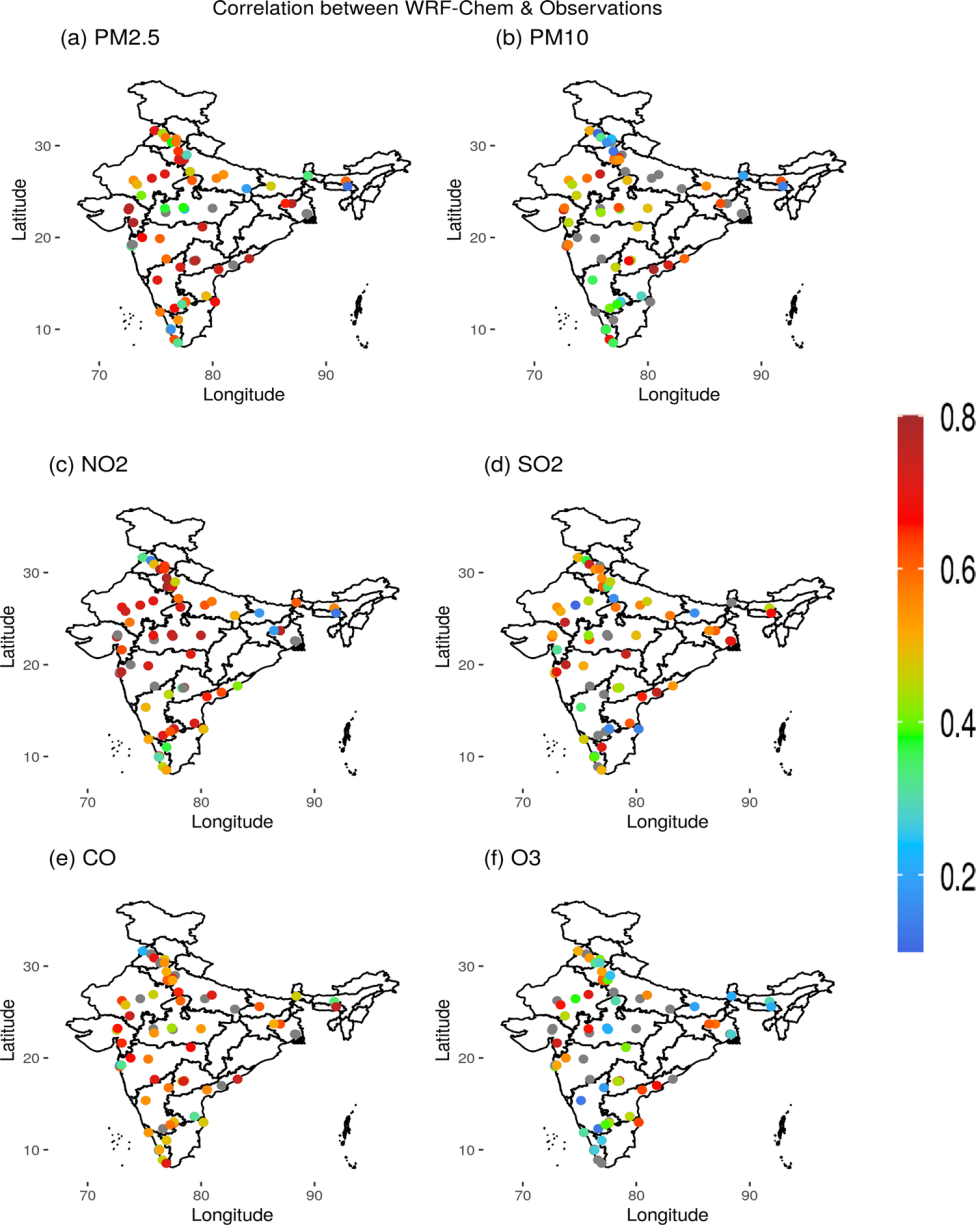
Correlation between CPCB data and WRF-Chem model outputs in (**a**) PM_2.5_, (**b**) PM_10_, (**c**) NO_2_, (**d**) SO_2_, (**e**) CO and (**f**) O_3_ obtained during 1 March 2020 to 30 April 2020. Gray color circles represents the locations that are not accounted for validation due to poor quality of the data. The figures are plotted using GrADS V2.2.1 software (http://cola.gmu.edu/grads/).

We further compared different pollutants concentrations (PM_2.5_, PM_10_, NO_2_, SO_2_, CO and O_3_) simulated by WRF-Chem with observations from different geographical locations across India collected by the Continuous Ambient Air Quality Monitoring Stations (CAAQMS) (https://app.cpcbccr.com/ccr/#/caaqm-dashboard-all/caaqm-landing), which are maintained by the Central Pollution Control Board (CPCB). The correlation coefficients between WRF-Chem and CPCB data for the above-mentioned pollutants during 25 March to 1 May 2020 at 71 locations, varied between 0.4 and 0.8 (at 95% confidence level) except very few locations (Fig. 2). A correlation coefficient of about ~ 0.7 is achieved at most of the locations and for all the pollutant concentrations except for O_3_ (~ 0.3). The correlation for NO_2_ and PM_2.5_ are reach their maxima of about ~ 0.8 over northwest, central and south India. Note that we have configured the scale factor to reduce the emissions uniformly across India by 40% during lockdown in the WRF-Chem simulation even though spatial variations in the reduction of these pollutants was reported based on ground^45^ and space borne measurements^44^. WRF-Chem simulated and the observed AODs at three AERosol RObotic NETwork (AERONET) stations, Gandhi College, Kanpur and Lahore, also suggest good correlations (at 95% confidence level) of about 0.67, 0,62 and 0.54, respectively (Figure S2).

We further computed the correlation coefficients at each grid point between observed tropospheric mean NO_2_ and SO_2_ in the planetary boundary layer from OMI and the corresponding WRF-Chem outputs during 1 March 2020 to 1 May 2020 (Figure S3), and presented those between MODIS AOD and WRF-Chem AOD in the same figure. In general, the WRF-Chem simulated AOD, NO_2_ and SO_2_ over the Indian region are significantly (at 95% confidence level) correlated with the satellite measurements. The WRF-Chem simulated NO_2_ and OMI derived NO_2_ exhibit reasonably good correlation (about ~ 0.6) over the Indian continent except over central India and the IGP region. The correlation values for SO_2_ are close to ~ 0.6 over the central and northwest parts of India. Similarly, WRF-Chem simulated and MODIS derived AOD also show a very good correlation of about ~ 0.8 over the entire Indian region. This gives further confidence that WRF-Chem is able to capture the variability of these pollutants reasonably well when compared to the ground and satellite-based measurements.

The spatial distribution of the AOD and trace gases (NO_2_ and SO_2_) predicted by WRF-Chem are compared with the satellite measurements in Figure S4. The composite mean of instantaneous values of AOD from swath data of MODIS and corresponding values of WRF-Chem obtained during March 8 and April 20, 2020 (Fig. S4a,b) indicate almost similar spatial distributions as those MODIS AOD over Central India, the IGP, and Northeast India, with lower values over Northwest India. The tropospheric mean NO_2_ and SO_2_ concentrations in the planetary boundary layer derived from the OMI (Fig. S4c,e) and the model (Fig. S4d,f) between March 8 and April 20, 2020 suggests that the NO_2_ and SO_2_ hotspots of slightly different magnitudes over Central and Northeast India. In summary, WRF-Chem is clearly able to simulate the gross spatial features over India and adjoining regions, albeit with slight differences in the magnitude.

The spatial distributions of MODIS AOD over India and the adjoining regions during PLD and DLD (Fig. S5a,b) exhibit higher concentrations of AOD (> 0.5) during PLD over the IGP (covering Punjab, Haryana, Uttar Pradesh, Uttarakhand, Bihar, and Central India) and a relatively clean atmosphere is observed over Northwest and South India. In contrast, a large increase in the AOD was observed over the head of the BoB, which persisted throughout the DLD. Surprisingly, an increase in the AOD (> 0.5) was observed over Central India and the BoB during DLD. The percentage difference in the AOD computed between the PLD and DLD periods (Fig. S5c) reveals a decrease (increase) of approximately 50–60% in the AOD over the IGP region (Arabian Sea, Central India, and BoB). The spatial distributions in the AOD obtained with WRF-Chem during PLD and DLD show similar distributions to those of MODIS (Fig. S5d,e), except for minor differences in the magnitude (Fig. S5f). Furthermore, the model shows an increase in the AOD over the entire Southern India including Arabian Sea while MODIS show reduced AOD over the southern part of India. Amnuaylojaroen et al.^49^ and Adedeji et al.^50^ stressed the need for a high resolution and improved emission inventory in order to obtain more accurate simulations of AOD over these regions.

NO_2_ is mainly produced by anthropogenic activities such as the combustion of fossil fuels and production of power. A drastic reduction in NO_2_ levels was therefore expected during the lockdown. The spatial distribution of NO_2_ from OMI during PLD is shown in Fig. S6a, with several noticeable hotspots of NO_2_ concentrations, over North and Northeast India. The intensity of most NO_2_ hotspots is decreased during the DLD period (Fig. S6b), following the reduction in fossil fuel burning and the significant reduction in the NO_2_ emissions from thermal power plants in Northeast India. A decrease of approximately 50–60% in NO_2_ levels is observed over both Northwest India and the IGP region, over the PLD and DLD periods (Fig. S6c). A similar percentage increase is also observed in Central India. Apart from a change in the magnitude of NO_2_ over the thermal plants, no significant changes were noticeable in the NO_2_ simulations over India (Fig. S6d,e). Increased NO_2_ concentrations were further observed in both the satellites observations and the model simulations over the Arabian Sea and the BoB.

The various data comparisons clearly suggests that WRF-Chem is able to reproduce well the observed aerosol and trace gas distributions, supporting the use of its outputs for qualitative analysis in the absence of observations. Because direct information about other trace gases (NO, NO_3_, N_2_O_5_, CO, O_3_, CH_4_, and SO_4_) and aerosol parameters (PM_2.5_, PM_10_, and hydrophilic and hydrophobic BC and OC) are not available from satellite measurements, the WRF-Chem simulations were analyzed to investigate the variability of these parameters during the lockdown.

### Changes in aerosol parameters during lockdown

**Figure 3:**
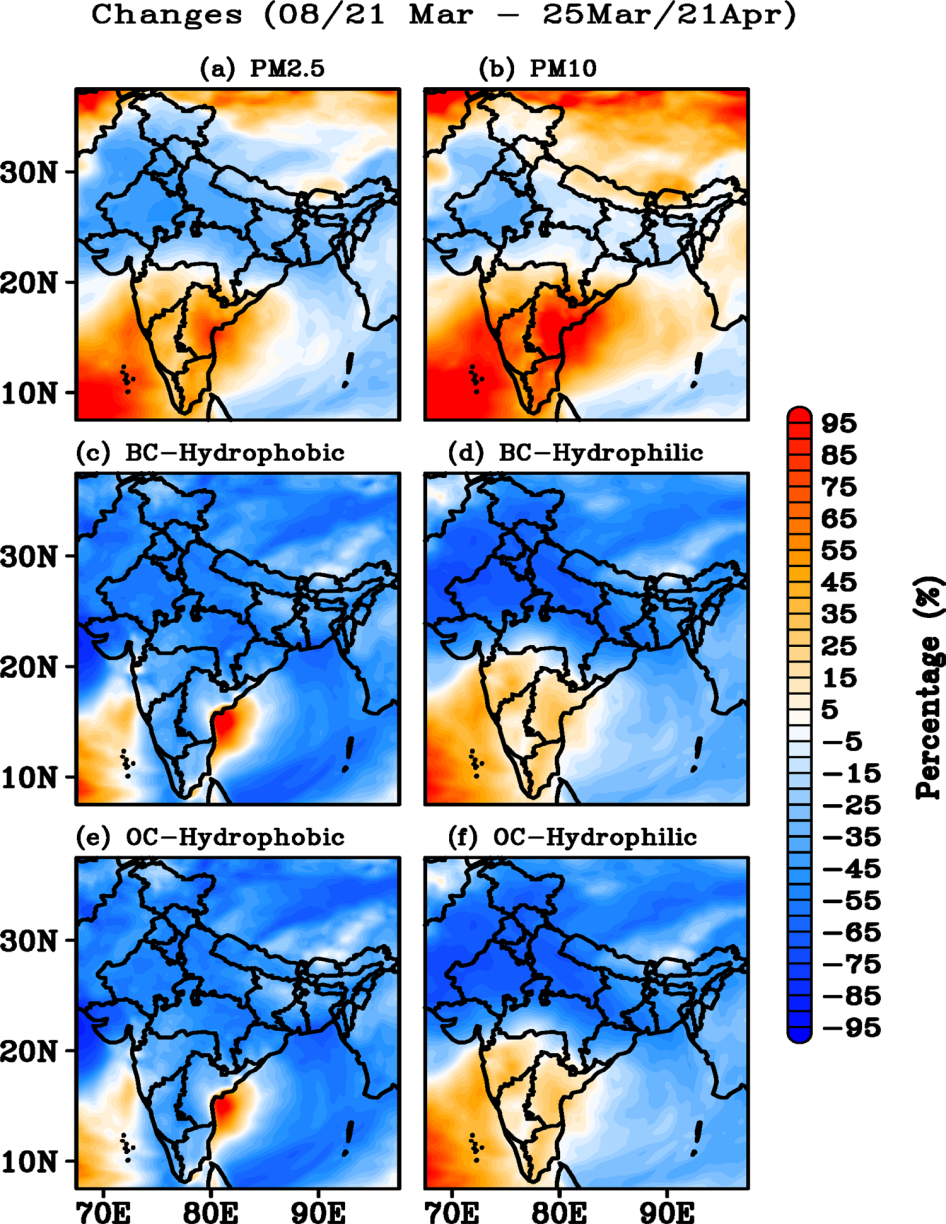
Percentage difference in the (**a**) PM_2.5_, (**b**) PM_10_, (**c**) BC hydrophobic, (**d**) BC hydrophilic, (**e**) OC hydrophobic, and (**f**) OC hydrophilic observed between the PLD and DLD periods based on WRF-Chem simulations for India and adjacent regions. The figures are plotted using GrADS V2.2.1 software (http://cola.gmu.edu/grads/).

The simulated particulate matter (PM_2.5_ and PM_10_) concentrations over India and the adjacent regions during DLD (Fig. 3) indicate a decrease of approximately 45–55% (15–25%) over Northwest India (IGP and Northeast India) and an increase of approximately 50–80% over South India (Fig. 3a,b) as compared to those during PLD. The BC produced in the atmosphere is generally hydrophobic (non-absorbing), but can be also hydrophilic (coated with water molecules)^51^ as further discussed at later stage. A decrease of approximately 45–55% in hydrophobic BC was observed over the Northwest, Northeast, and IGP regions, with a slight enhancement over South India, the Arabian Sea and BoB (Fig. 3c) during DLD, compared to PLD. The hydrophilic BC concentrations decreased by approximately 60–70% over the northwest, northeast, and the IGP regions, while an increase of 35–45% was observed in South India during DLD (Fig. 3d). Similar changes in the hydrophobic and hydrophilic OC concentrations (Fig. 3e,f) were observed over India. The observed large reduction in hydrophilic BC compared to hydrophobic BC is mainly due to its representation in the percentage change. However, the magnitudes of hydrophobic and hydrophilic BC and their differences indicate (Fig. S7) that both components were reduced in similar magnitudes during the lockdown period. Further, as expected, high magnitudes of hydrophobic BC are also noticeable.

Since seasonal changes during the PLD and DLD periods may contribute to the observed features, we have performed an additional simulation for the year 2019 using WRF-Chem, considering the same PLD and DLD for the analysis. While the simulated particulate matter (PM_2.5_ and PM_10_) concentrations over India during DLD (2020) show (Fig. S8) no significant change over Northwest India, a significant decrease in PM_2.5_ (35–45%) over IGP and Northeast India and 15–25% increase over South India (Fig. S8a,b) are noticeable when compared to 2019, whereas PM_10_ slightly increased (25–35%) throughout India in 2020 compared to 2019. A decrease of approximately 45–55% in hydrophobic BC was observed throughout India, with a slight enhancement over the BoB (Fig. S8c) during 2020, compared to 2019. The hydrophilic BC concentrations decreased by approximately 60–70% over the northwest, northeast, and the IGP regions, while an increase of 25–35% was observed in South India during 2020 (Fig. S8d). Similar changes in the hydrophobic and hydrophilic OC concentrations (Fig. S8e,f) were observed during 2020 compared to 2019. This clearly suggests that the seasonal changes are relatively small compared to the observed differences in the aerosol concentrations during the DLD period.

### Changes in trace gases concentrations during lockdown

**Figure 4:**
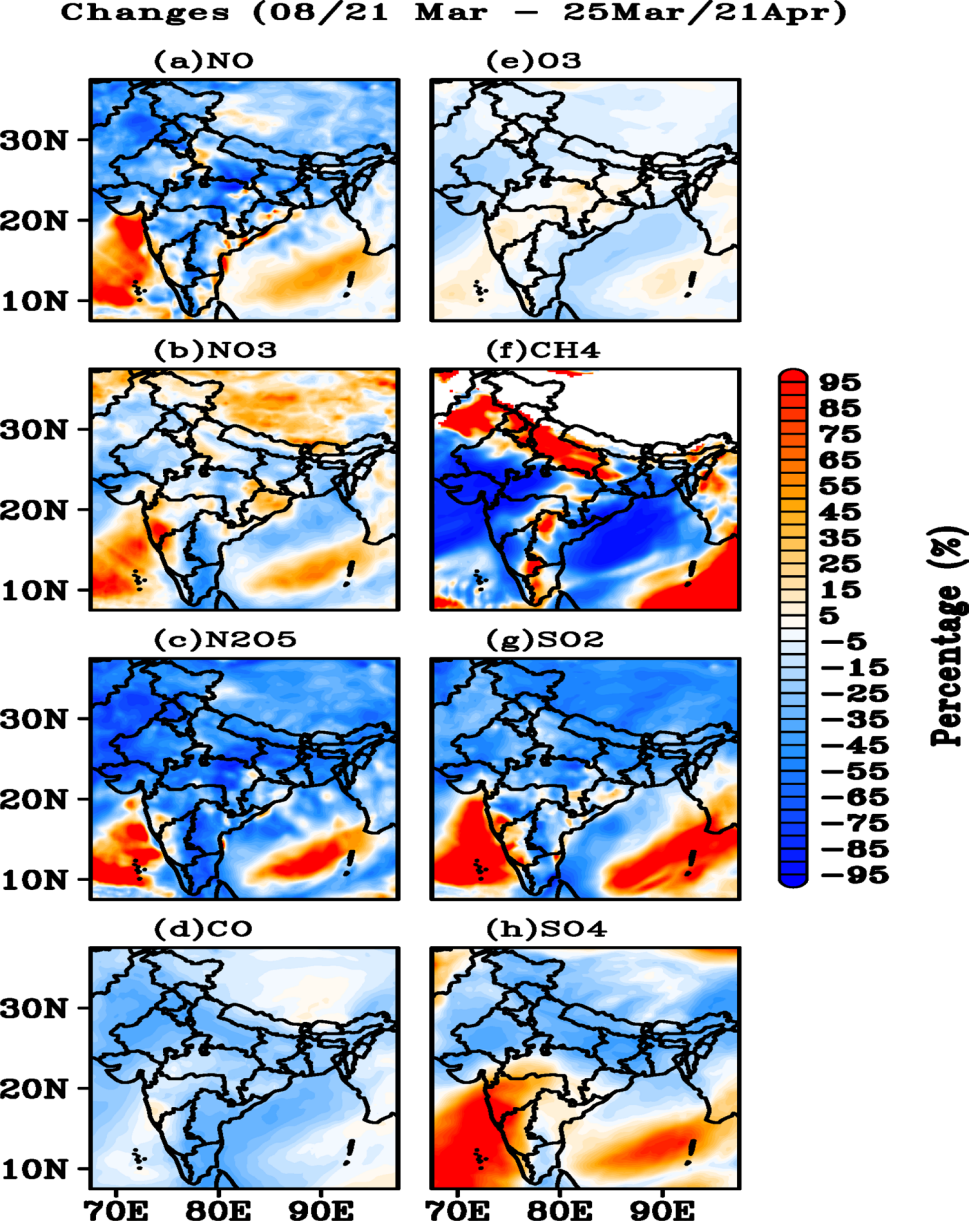
Percentage difference in the (**a**) NO, (**b**) NO_3_^−^, (**c**) N_2_O_5_, (**d**) CO, (**e**) O_3_, (**f**) CH_4_, (**g**) SO_2_, and (**h**) SO_4_^2−^ observed between the PLD and DLD periods based on WRF-Chem simulations for India and adjacent regions. The figures are plotted using GrADS V2.2.1 software (http://cola.gmu.edu/grads/).

The simulated concentrations of trace gases over India and the adjacent regions suggest (Fig. 4a) a decrease of approximately 45–55% in the NO concentrations over most parts of India, an increase of approximately 25–35% over the IGP region with a few hotspots, and an increase of approximately 55–65% over the BoB and Arabian Sea during DLD as compared to PLD. The simulated NO_3_ concentrations show a decline of approximately 25–35% over Rajasthan, Gujarat, and Haryana during DLD (Fig. 4b). A sharp increase of approximately 55–65% (25–35%) in NO_3_ concentrations is also noticeable over the south west of India and Arabian Sea (IGP). A clear decline of approximately 65–75% in the N_2_O_5_ concentration is observed over the IGP, Northeast, and Northwest India, in contrast with an increase of similar magnitude over Arabian Sea and BoB during the DLD (Fig. 4c).

A decrease of approximately 15–25% in the concentration of CO was observed over many parts of India, while CO increased by approximately 5–15% over western part of Maharashtra during DLD (Fig. 4d). The simulated O_3_ concentrations increased by 25–35% over Arabian Sea, Central India, and a decrease of a similar magnitude is noticeable over Northwest and North India (Fig. 4e). Pronounced increase in CH_4_ concentrations (65–75%) is observed over IGP and some parts of Central and south India during DLD, despite a sharp decrease (65–75%) over Rajasthan and Gujarat including a few hot spots crossing from south to northeast India (Fig. 4f). The SO_2_ levels decreased by 45–55% over several parts of India with a few hotspots in Southwest India, and 75–85% increase over the Arabian sea during the DLD (Fig. 4g). Surprisingly, the SO_4_ levels increased by 80–90% between the Arabian sea and the central parts of India (35–45%) and the BoB, while simultaneously decreasing over Northwest and Northeast India during DLD (Fig. 4h).

The analysis of WRF-Chem for the year 2020 suggests a significant decrease (45–75%) in all concentrations (except CH_4_ and SO_4_) during the DLD period when compared to the same days during 2019 (Fig. S9). O_3_ also shows a reduction throughout India in 2019 unlike during 2020 (Fig. S9e). An increase in SO_4_ concentrations over central and southern India including BoB with slight decrease over IGP is noticeable (Fig. S9h). This again suggests that the seasonal changes are small compared to the observed differences in the trace gases concentration during the DLD period.

In general, freshly emitted BC and dust aerosols will be hydrophobic and inert. After the aging process, for which time varies from 1.6 to 2 days depending on the pollutant loading and dynamics^52^, they may become hydrophilic after mixing with the inorganic and organic acidic species. Hydrophobic‐to‐hydrophilic conversion is controlled by their interaction with more hydrophilic species such as sulphates, nitrates, and secondary organic aerosols^52^. Upon emission, all BCs are assumed to be in the insoluble Aitken mode (mean radius 0.03 μm), and the subsequent aging and growth explicitly depends on the ambient concentrations of sulphate^53^.

Despite the significant reduction in anthropogenic emissions during the lockdown, however, the emissions never reduced to zero (practically impossible) because of essential services (power plants, food industries, health care system, essential transport, agricultural activities etc.). Additionally, there were also natural emissions from the biogenic sources, biomass burning, agriculture activities, and forest fires (Fig. S10 shows the MODIS fire counts). Moreover, the observed reductions were not uniform in all the regions (Figs. 3, 4), indicating some active anthropogenic emissions in certain regions (for example central and southern India). Atmospheric dynamics due to the formation of anticyclonic condition over central India also played a crucial role in the observed pollutants (Fig. S12). The results suggest an accumulation of pollutants in this region, suggesting that despite the reduction in the anthropogenic emissions during the lockdown, there was a continuous accumulation of pollutants in the central India likely associated with the dynamical conditions. Once the pollutant concentration increases, the active chemical conversions take place and forms the acidic species such as sulphates, nitrates and organic acids (this is clear from Fig. 4b,h, which show a substantial increase in the acidic species such as sulphates and nitrates).

The reduction in NO_x_ resulted in the increase of O_3_ concentration (lack of O_3_ scavenging by NO, Fig. 4a). There was also an increase in the relative humidity (RH) (Fig. 6d) and solar irradiance^54^, which favors OH formation^55^. This should increase the oxidative processes (initiated by OH) of the trapped air parcel and result in the formation of sulphates and nitrates from the precursor species such as SO_2_, NO_x_, and NH_3_^56^. Some reports suggested that the increase in carbonaceous aerosols resulted from VOC oxidations initiated by O_3_ as O_3_ concentrations increase during the lockdown^57^. Therefore, accumulation of pollutants and subsequent oxidative processes under favorable conditions increased the acidic species and upon mixing with BC and dust, led to their conversion into hydrophilic species. The increased RH may have increased the size of these species, which resulted in higher AOD.

### Changes in the vertical distribution of aerosols during lockdown

The analysis included a comparison of the percentage changes in the near-surface-level aerosol and trace gases concentrations during DLD with those during PLD. Despite the observed clean atmosphere over India^15,32^, the concentration of aerosols increased over Central India during DLD, which affected the AOD. An increase in the concentration of other aerosols (PM_2.5_, PM_10_, and hydrophilic BC and OC) was also observed over Central and South India despite the absence of local pollution sources at the surface during the DLD. Because the AOD is an integrated parameter that reflects aerosol extinction across the column, the increase in the AOD should be related to higher aerosol concentrations in the column.

**Figure 5:**
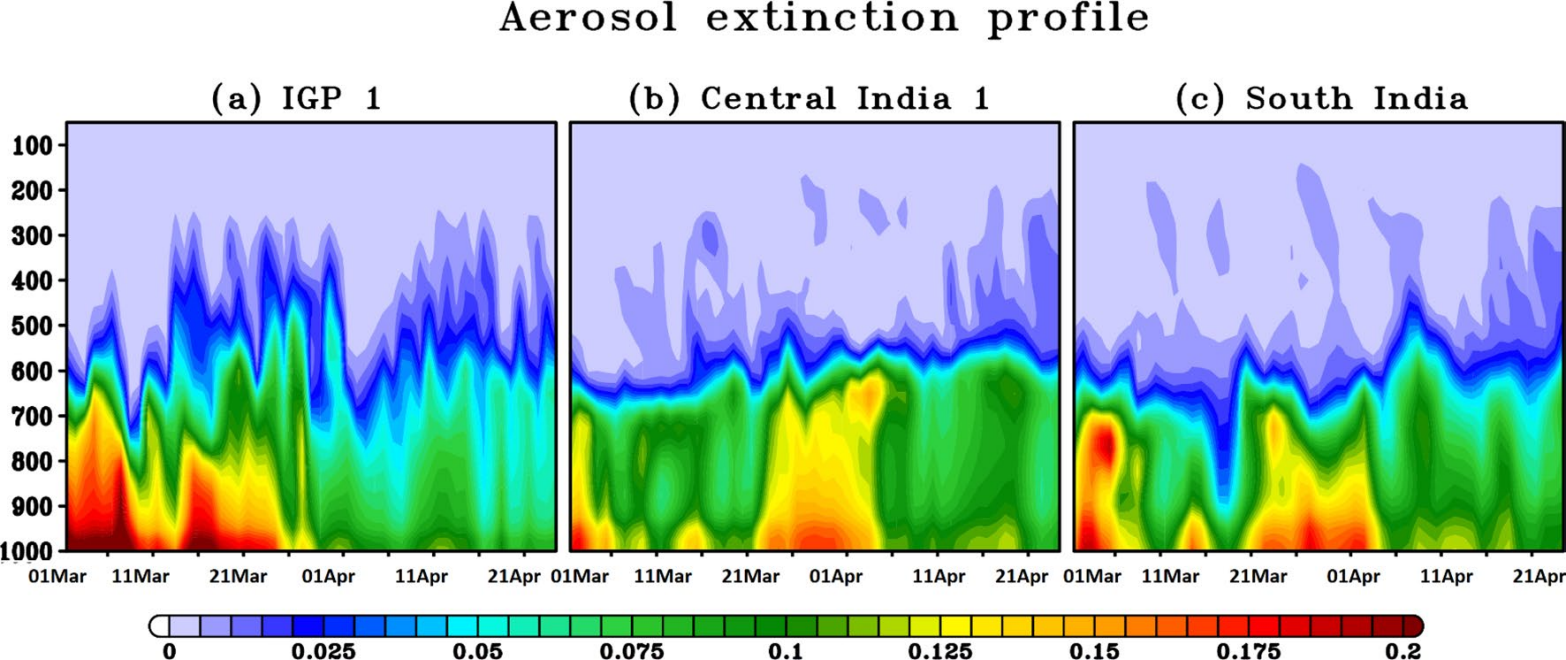
Vertical distribution of the aerosol extinction coefficient observed over (**a**) the IGP region, (**b**) Central India, and (**c**) South India during PLD and DLD periods. The figures are plotted using GrADS V2.2.1 software (http://cola.gmu.edu/grads/).

Figure 5 outlines the vertical distribution of the aerosol extinction coefficient over selected regions in the IGP (Fig. 5a), Central India (Fig. 5b), and South India (Fig. 5c) during the PLD and DLD periods. The high (> 0.15 km^−1^) vertical aerosol extinction that was observed over the IGP during the PLD period reduced drastically (< 0.1 km^−1^) during DLD. This is expected as the complete lockdown reduced the emission of anthropogenic aerosols from traffic and industrial activities. Interestingly, a low extinction coefficient was observed (< 0.1 km^−1^) during PLD over Central and South India, whereas the extinction (> 0.15 km^−1^) was observed to increase from the surface and 600 hPa with a slight decrease between the two, particularly over Central India. The elevated aerosol layers, particularly over Central India, might have contributed to the observed increase in AOD during the DLD period. The possible reasons behind this increase are discussed in the following Section.

## Discussion

The lockdown that was enforced in India between March 25 and April 14, 2020 (Phase 1) due to the COVID-19 pandemic restricted the movement of people and constrained all businesses and industrial activities, which helped in the improvement of the air quality. Our analysis indicates that certain parts of India (the northwest, the IGP, and the northeast) enjoyed a clean environment in line with the results of studies by Jain and Sharma^15^ and Sharma et al.^32^. However, despite the absence of anthropogenic activities at the surface during the lockdown, higher aerosol and some trace gases concentrations were observed in some parts of India (Central and South India), which suggests that other factors might have contributed to the increased concentrations, including emissions from natural sources (such as forest fires, biomass burning) and long-range transport.

The fire radiative power (FRP) obtained from the MODIS measurements as a proxy for biomass burning over India and the adjacent regions during PLD and DLD is shown in Fig. S10. Note that the first phase of DLD is again divided into two periods (March 25 to April 7, 2020 and April 8–20, 2020), as both the model simulations and the observations show an increase in the concentration of aerosols and trace gases during the first week of April 2020 (Fig. 1). An FRP of approximately 5–10 MW was observed over South India and the IGP region during the PLD period, with a relatively weaker FRP observed over Central India. A few hotspots in which the FRP was particularly high were observed over Northeast India. An increase in FRP was observed over Central India and the IGP regions, with small changes in other parts of India during DLD. A relatively high FRP was observed over Central India during the second half of the DLD (April 8–20, 2020) as compared to the first half (March 25 to April 7, 2020). However, the IGP region remained clear. These intermittent fire activities might have contributed to the observed increase in some trace gases (Fig. 4) and aerosol (Fig. 3) concentrations over Central and South India during DLD.

**Figure 6:**
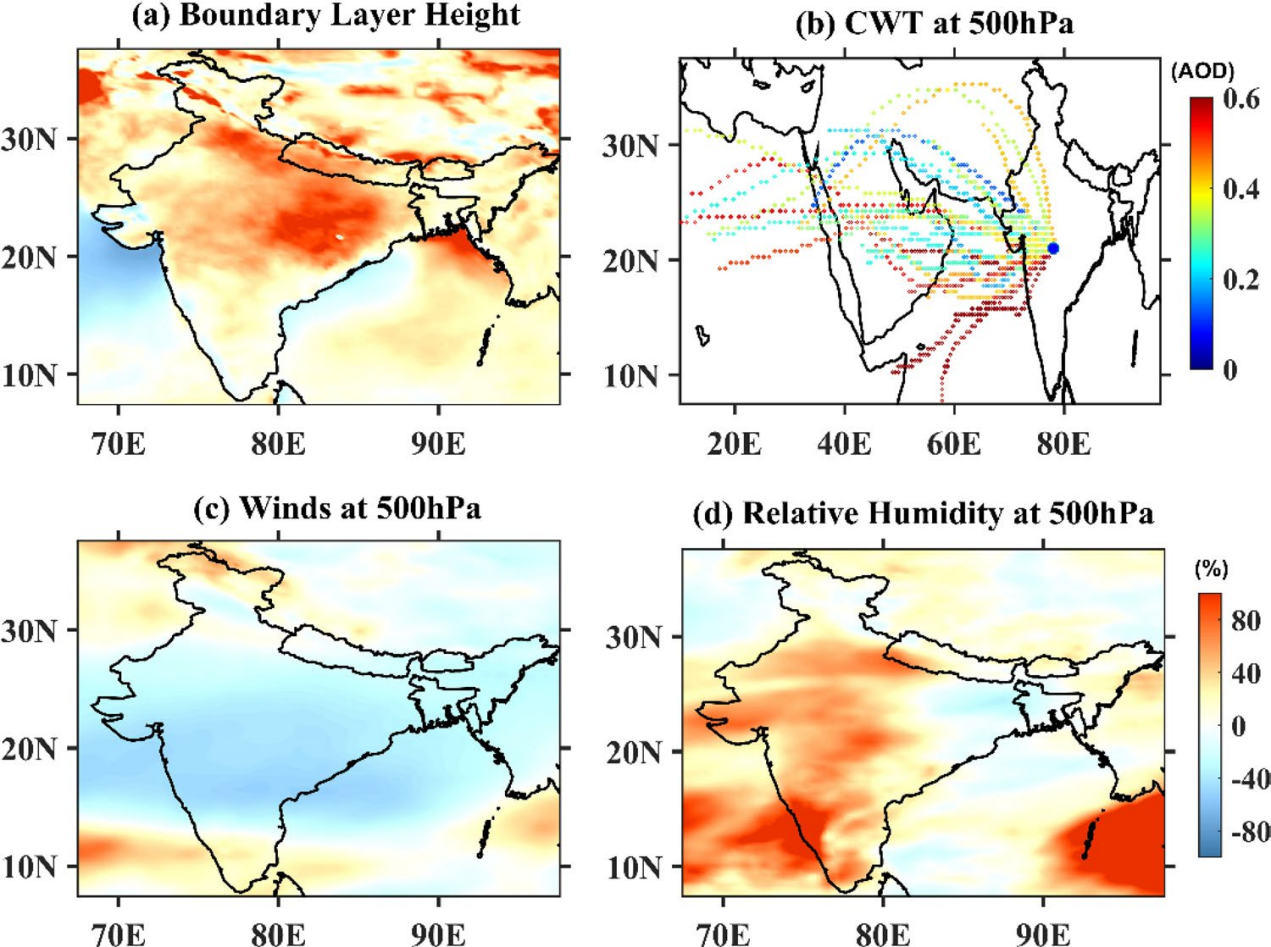
Percentage difference in the (**a**) BLH, (**c**) wind speed at 500 hPa, and (**d**) RH at 500 hPa observed between the PLD and DLD periods based on WRF-Chem simulations for India and the adjacent regions. (**b**) Concentration weighted trajectory (CWT) map of the AOD for Central India during the DLD period (March 25–April 20, 2020). The blue dot denotes the center of central India. The figures are plotted using GrADS V2.2.1 software (http://cola.gmu.edu/grads/).

The implemented lockdown (first phase) over India falls during the transition period between winter and summer, during which the increase in the boundary layer height (BLH) leads to strong vertical mixing, affecting the aerosol and trace gas concentrations at the surface. An increase of approximately 70–80% in the BLH occurred (Fig. 6a) over the IGP and Northeast India between the PLD and DLD periods, compared to a 5–25% increase over the rest of India. This may have also been a factor behind the significant reductions in the aerosol and trace gas concentrations near the surface alongside the low emissions. The southern parts of India experienced negligible changes in the BLH between the PLD and DLD. Very similar changes in BLH are also observed in ERA-5 reanalysis (Fig. S11).

A significant decrease (increase) in BLH is noticeable over south and central India (IGP and northeast) during 2020 compared to 2019 (Fig. S11c). Besides low emissions, the increase in BLH over IGP and north India may have resulted in the reduction of the pollutants due to well mixing. Similarly, an increase in these pollutants (particularly aerosols) over central and south India due to the decrease (or significant reduction when compared to 2019) in the BLH. We have also retrieved the boundary layer altitude using the network of ground-based radiosonde measurements over the Indian region to validate the WRF-Chem simulated boundary layer heights (Fig. S11d). WRF-Chem seems to overestimate the variations in BLH compared to radiosonde data. Despite the changes in the magnitudes between the WRF-Chem and radiosonde observations, the increase in the BLH by 20–40% (10–20%) in the IGP and northeast region (south and central India) is noticeable in 2020, compared to 2019. The increase in BLH in 2020 may be one of the reasons for the decrease in pollutant concentrations in 2020 in addition to the reduced emissions. However, the percentage reduction in concentrations does not go hand-in hand with the BLH changes as the pollutant concentrations cannot interact linearly with the BLH but several other factors such as mixing efficiency, wind speed (both vertical and horizontal), topography etc., also influence the pollutant concentrations.

In contrast to the decrease in aerosol concentrations, a significant increase in the AOD was observed both by the satellite and in the model simulations over Central and South India during the lockdown. The dynamics and background meteorology might have influenced the high AOD observed over these regions. The dry season, together with high winds, favors the production and transport of dust which contributes to the AOD, especially over Central India. The mean wind patterns averaged over the DLD period (at 850, 700, and 500 hPa in Fig. S12), indicate that the direction of the wind reversed in middle-eastern Africa, where the largest source of desert dust is located. Dust transported from these regions got trapped in the anti-cyclonic circulation, as indicated by the wind vectors between 500 and 300 hPa (figure not shown) over Central India and the head of the BoB.

To further investigate the potential transport of aerosols over large geographical scales, Concentration Weighted Trajectory (CWT) maps of the AOD in Central India were investigated. The CWT includes atmospheric concentrations combined with back-trajectories and information about residence times and can identify the air parcels that may be responsible for the high concentrations observed over a given region^23,58^. To identify the transport pathways of aerosols, 72-h back trajectories were calculated for 850, 700, and 500 hPa. The calculations were carried out at 06.00 h (UTC) during the DLD period (March 25 to April 14) using a 0.25° × 0.25° grid. The CWT analysis of the AOD suggests (Figs. S13 and 6b) that the sources of the observed aerosol levels are located in Africa. Except for the lower pressure level (850 hPa), air pathways from the southeast contribute to the observed AOD (Fig. S13c), and all the trajectories are long-range (Fig. S13a,b). Thus, the elevated aerosol layers seen in Fig. 5 are the result of long-range transport over Central India that might have get trapped in the anti-cyclone (Fig. S12a), despite the decrease in particulate matter near the surface during lockdown.

**Figure 7:**
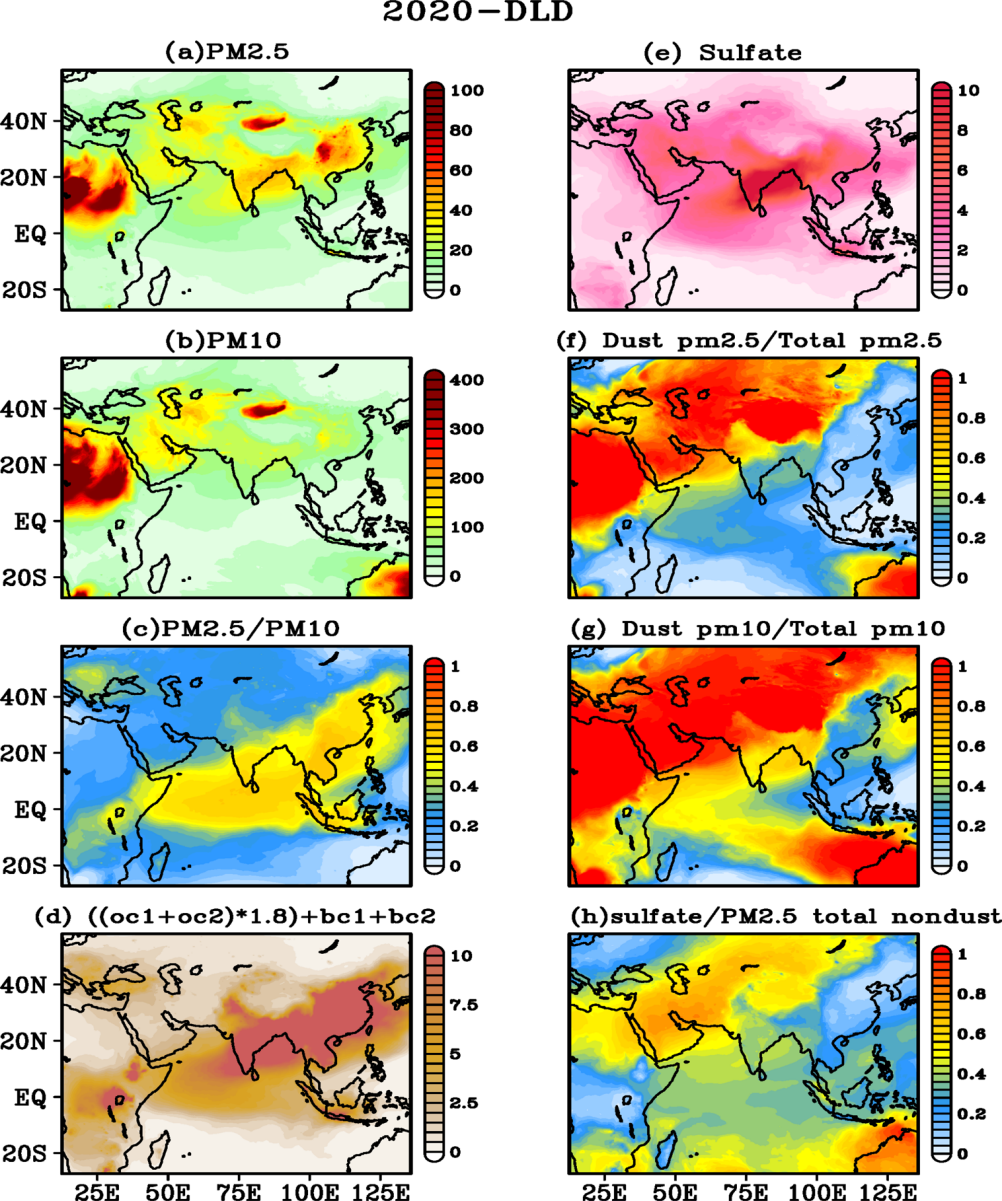
Spatial distribution of (**a**) PM_2.5_, (**b**) PM_10_, (**c**) ratio between PM_2.5_ and PM_10_, (**d**) organic matter and black carbon ((OC hyrdrophobic + OC hydrophilic) × OC mass fraction (1.8) + BC hyrdrophobic + BC hydrophilic), (**e**) sulfate, (**f**) ratio between dust PM_2.5_ and total PM_2.5_, (g) ratio between dust PM_10_ and total PM_10_, and (**h**) ratio between sulfate and PM_2.5_ total non-dust obtained during DLD period based on WRF-Chem simulations for India and adjacent regions. All units are in μg m^−3^ except ratios. The figures are plotted using GrADS V2.2.1 software (http://cola.gmu.edu/grads/).

To further assess the contributions from local and long-range transport, we show a wider spatial distribution of aerosol pollutants in Fig. 7. PM_2.5_ (Fig. 7a) and PM_10_ (Fig. 7b) reveals high concentrations over the dust source regions of Africa and the middle-east. The ratio of PM_2.5_ and PM_10_ (Fig. 7c) is low (0.2–0.3) over these regions, suggesting that these are source regions where both fine and coarse particles are generated. Since coarse particles cannot be transported far from the source regions compared to fine particles due to their shorter lifetime (related to dry deposition processes), higher ratio values (0.5–0.7) are observed over the south and central regions of India and farther from the main dust sources. The sum of combined surface concentrations of organic matter and black carbon show much smaller values (Fig. 7d). Sulfate concentrations are found to be high (Fig. 7e) over the south and northeast parts of India where thermal power plants are located. Contributions of dust to PM_2.5_ and PM_10_ calculated as their ratio’s further show (Fig. 7f,g) high contribution (> 80%) near the dust sources, and relatively low contribution (30–40%) over Indian region, particularly over the south and northeast parts of India. The ratio between the concentration of sulfate aerosol with respect to the total concentration of PM_2.5,_ suggest that non-dust aerosols are relatively low (< 0.45) over India during lockdown (Fig. 7h). Similar features are also noticed during the same period of the year 2019, suggesting that long-range transport of aerosols dominates over India during this season.

To further investigate the role of meteorology and dynamics in the observed increase in aerosol concentrations over Central India, differences in the wind speeds at 850, 700, and 500 hPa averaged during the PLD and DLD periods are shown in Fig. S14. The difference in the wind speeds is very small at 850 hPa but increases at higher levels. An increase of approximately 80–90% in wind speed can be seen over IGP region, South India, which decreases to 50–60% over central India during DLD. A decrease of approximately 50–60% is also observed in the wind speed at 500 hPa, particularly over central India during DLD (Figs. 6c and S14c). These reduced wind speeds mean that pollutants remain longer in central India than they do in the north.

The percentage changes in the relative humidity between the PLD and DLD periods suggest (Fig. S14d–f) an increase of approximately 70–80% during lockdown over India, particularly at 500 hPa (Figs. 6d and S14f). This increase in the relative humidity increases the size of aerosol particles, leading to a higher AOD^59^, as reflected in the increase in hydrophilic BC and OC (Fig. 3). Meteorology and dynamics have therefore played an important role in the observed increase in AOD over Central India during the lockdown.

## Conclusions

Observations based analyses have shown that lockdown measures implemented to combat COVID-19 has led to significant improvement in air quality over India, particularly during the first phase of the lockdown (i.e., March 25 to April 14, 2020). WRF-Chem model simulations also revealed similar findings. WRF-Chem outputs were then analyzed to understand the processes driving the aerosols and trace gas concentrations variability across India and adjoining regions. Our results revealed that an increase in boundary layer altitude (well mixing) together with an enhancement of the wind speeds (dispersion) played major roles in the observed clean atmosphere in the north (and northeast) India besides the low emissions during the lockdown. At the same time, we demonstrated that an elevated aerosol layer (above 600 hPa), through long-range transport, a decrease in background wind speed (stagnant condition), an increase in relative humidity (hygroscopic), and no significant change in the boundary layer altitude are the main reasons behind the observed increase in the aerosol optical depth during lock down over Central India^44^.

Several recent reports^60^ investigated the percentage changes in pollutants due to the reduced mobility of the traffic during COVID-19 lockdown. A Mobility index was determined from the mobile phone usage at the country level, which indirectly suggests the human movement. The strong correlation between the decrease in NO_2_ concentrations and decrease in the mobility index was clear. However, such relation is not found in the particulate matter (PM_2.5_), suggesting that PM_2.5_ changes are not directly related to human mobility^60^. To illustrate the gross traffic volume changes over India, we have considered Google^61^ Community Mobility Report (CMR) data based on the previous traffic associated pollution studies during the COVID-19 lockdown. This CMR parameter is computed from the baseline value (median) available for the corresponding day of the week (during COVID-19 lockdown period) from the values prevailed during the 5-week period of study (data period between 3 January and 6 February 2020). The CMR data indicates a clear reduction in the mobility (at supermarkets and pharmacy (− 16%), retail and recreation (− 56%), public transport (− 41%), workplaces (− 33%) and park (− 34%) categories) during the lockdown across India with some spatial variations. However, there was an increase of 20% in the mobility in the residential areas. Though one-to-one relation cannot be obtained (as these mobility statistics from mobiles do not fully represent the actual scenario), similar reduction (50–60%) in the pollutants is clearly observed in NO_2_ and PM_2.5_ (and PM_10_) in the north-west and IGP including the north-eastern parts. However, such relation is not found over the central India, suggesting the complex nature of these pollutants influenced by the background meteorology and dynamics.

It is therefore prudent to conclude that large-scale meteorology and dynamics play an important role in the changes in pollution levels over India and the adjacent regions with respect to the lockdown. The measures implemented by the Indian government to reduce pollution levels should also consider these facts in their decision making. COVID-19 provided an opportunity to test this hypothesis by ceasing all major anthropogenic activities, providing the background for a large-scale natural laboratory experiment.

## Supplementary Information

Supplementary Information.
