# Supplementary material for: Natural processes dominate the pollution levels during COVID-19 lockdown over India
Source: Sci Rep. 2021 Jul 23;11:15110. doi: 10.1038/s41598-021-94373-4 (PMC8302761; doi:10.1038/s41598-021-94373-4)
Supplement: Supplementary file 1 — Supplementary Information. [file 41598_2021_94373_MOESM1_ESM.docx]

Title page:

**Natural processes dominate the pollution levels during COVID-19 lockdown over India**

Venkat Ratnam Madineni^1^_,_ Hari Prasad Dasari^2^, Rama Krishna Karumuri^2^, Yesubabu Viswanadhappli^1^, Prasad Perumal^1^, and Ibrahim Hoteit^2,*^

^1^National Atmospheric Research Laboratory, Gadanki, Andhra Pradesh, India

^2^ Physical Sciences and Engineering Division, King Abdullah University of Science and Technology, Thuwal, Saudi Arabia

*Corresponding author:

Prof. Ibrahim Hoteit

Physical Sciences and Engineering Division,

King Abdullah University of Science and Technology, Thuwal, Saudi Arabia

E-mail: ibrahim.hoteit@kaust.edu.sa

**Supplemental Material:**

**S1 Details of the experimental setup and datasets**

**S1.1 Model design**

WRF-Chem [1] was configured with 90 vertical levels extending up to a height of 10 hPa and a horizontal resolution of 30 km covering both Asia and the regions around the Indian Ocean (Fig. S1). The arc 5 min (∼10 km) terrain information from the United States Geological Survey (USGS), and the land use, land cover, and soil information were taken from Moderate Resolution Imaging Spectroradiometer (MODIS). The model was integrated from 00:00 UTC on February 20 until 00:00 UTC on May 01, 2020. The first 15 day of the simulation were treated as spin-up and thus excluded from the analysis. The remaining period over the different phases of lockdown in India was used for the analysis. The model initial conditions were extracted from the Final reanalysis (FNL) data, which are available at a 1° × 1° spatial resolution, and the time-varying high-resolution sea surface temperature are taken from the real-time global high-resolution data [2] to update the lower boundary conditions. The initial and boundary chemical conditions are taken from the Whole Atmosphere Community Climate Model dataset (WACCM) [3]. The meteorological and chemical conditions at the lateral boundary were updated every six hours. The following model physics was configured for atmospheric component which includes the Thompson scheme [4] for the cloud microphysical processes, the Yonsei University scheme [5] for the boundary layer process, the Kain–Fritsch scheme [6] for cumulus convection, the NOAH MP scheme [7] for the land surface processes, and the rapid radiative transfer (RRTMG) radiation scheme [8] for both longwave and shortwave radiation. Analysis nudging was applied throughout the simulation for the meteorological variables of zonal and meridional winds, temperature, and moisture of FNL fields every 6 h. The Model for Ozone and Related chemical Tracers (MOZART) scheme [9] was used for gas phase chemistry coupled with Goddard Chemistry Aerosol Radiation and Transport (GOCART) aerosol chemistry module, and the GOCART- Air Force Weather Agency (AFWA) [10, 11] dust scheme was used. Model of Emissions of Gases and Aerosols from Nature (MEGAN) scheme used for the calculation of online biogenic emissions [12] and the deposition of surface aerosols and gases were simulated using wesely dry deposition scheme [13].

Anthropogenic emissions were extracted from the Emission Database for Global Atmospheric Research (EDGAR) Hemispheric Transport of Air Pollution (EDGAR-HTAP-v2) for WRF-Chem simulations during the period of pre-lockdown. This emission data is prepared in 2010 by collecting the localized information from regional inventories to produce a global inventory of emissions. The EDGAR-HTAP has considered all major emission sectors, htap_1_air = international and domestic air, htap_2_shipping = international shipping, htap_3_energy = power industry, htap_4_industry = manufacturing, mining, metal, cement, chemical, solvent industry, htap_5_transport = ground transport (incl. road, rail, pipeline, inland waterways), htap_6_residential = heating/cooling of buildings and equipment/lighting of buildings and waste treatment, and htap_8_agriculture = agriculture but not agricultural waste burning. These emissions were mapped to the model grids using scaling factors based on the projections provided in Venkataraman et al. [14] for India region to represent the updated emissions scenarios during the period of pre-lockdown (i.e., from 08^th^ March 2020 to 24^th^ March 2020). We conducted sensitivity experiments to find out the realistic estimate of the percentage reduction in emissions from different emission sectors over India, the scale factors are modified through changing the percentage of emissions (from 30 to 70 %) from these sectors at a given time based on the recent COVID-19 observational studies [15,16,17,18] From WRF-Chem simulations, we found that an overall emission reduction of 40% as anthropogenic emission inventory is able to reproduce a realistic estimate of the observed concentrations during the COVID-19 lockdown. Further, we have utilized these scale factors (associated with 40% reductions) to represent the realistic changes due to the impact of COVID-19 in the anthropogenic emissions inventory for simulating WRF-Chem model during the lockdown period (i.e., 25^th^ March 2020 to 1^st^ May 2020). The chemical species as part of anthropogenic emissions includes CO, SO_2_, NO_x_, NH_3_, NMVOCs, BC, OC, PM_2.5_, PM_10_. Though these scaling factors used for the anthropogenic emissions inventory of WRF-Chem may not represent completely the real scenarios of emissions prevail during the COVID-19 lockdown, they provide the overall reduction indication to approximately account the changes associated with the lockdown. Further, it is also not possible at this time to prepare such actual inventory, as it needs a coordinate effort. Moreover, the initial and boundary conditions of chemical fields used for WRF-Chem plays significant effect on simulation of these chemical species as emissions inventory acts like a first layer or background. Moreover, in this study, to provide accurate feedback of Chemical initial conditions, we initialized the model during the lockdown with 5-day spin-up period using the WACCM initial conditions. This WACCM data base assimilates real-time all available observations of different chemical species [3].

**S1.2 Datasets**

We used various observational datasets to evaluate the WRF-Chem outputs and to explore the variability in the concentrations of aerosols and trace gases before and during the lockdown phases over India. The model AOD was validated against the instantaneous measurements of AOD MODIS C6.1 Level 3 (MOD08_D3) observations [19], which are available on a 1^o^ × 1^o^ grid (https://modis.gsfc.nasa.gov). The instantaneous measurements of tropospheric columnar nitrogen dioxide (NO_2_) and planetary boundary layer sulfur dioxide (SO_2_) are taken from the Ozone Monitoring Instrument (OMI) (http://disc.sci.gsfc.nasa.gov) and nadir-viewing spectrometer onboard NASA’s Aura satellite datasets were used to validate the WRF-Chem trace gas outputs.

Several well-calibrated trace gas datasets collected using cavity ring-down spectroscopy (CRDS) and Greenhouse gas analyzers that are available over Gadanki (13.5°N, 79.2°E) were used to validate the WRF-Chem outputs. Measurements of trace gases such as O_3_, NO_2_, NO, CO, and SO_2_ were obtained using HORIBA trace gas analyzers (Model APOA 370-O_3_, APNA 370-NO_x_, APMA 370-CO, and APSA 370-SO_2_) and calibrated using the traceable standards from the National Institute of Standards and Technology (NIST) [20]. The analyzers use the lower detection limits of 0.5, 0.5, 20, 0.5, and 0.4 ppb for O_3_, NO_2_, NO, CO, and SO_2,_ respectively [20].

Data generated by the seven-channel Aethalometer (AE31), which is continuously operated at NARL, Gadanki [21], was used to validate the WRF-Chem simulated black carbon (BC) concentrations. The AE31 measures BC concentrations in the range 0–500 μg/m^3^, with uncertainties ranging from 50% at 0.05 μg/m^3^ to 6% at 1 μg/m^3^ [22]. The direct and diffuse sky radiances collected from ground-based measurements using a sky radiometer (POM-01L, Make: Prede Co. Ltd, Japan) were used to retrieve the AOD at 5 wavelengths: 400, 500, 675, 870, and 1020 nm. The AOD at 500 nm was used to compare the optical properties of aerosols in the WRF-Chem simulations.

The daily averaged data of PM_2.5_, PM_10_, NO_2_, SO_2_, CO and O_3_ from 01 March – 01 May 2020 was acquired from Continuous Ambient Air Quality Monitoring Stations (CAAQMS) situated across India (https://app.cpcbccr.com/ccr/#/caaqm-dashboard-all/caaqm-landing), which are managed by Central Pollution Control Board (CPCB), Delhi. The quality of data was ensured by filtering the outliers and constant values. Missing records, erroneous data and outliers in the observation have been discarded for the analysis considering them as invalid data following method mentioned in Singh et al. [18]. This quality check resulted to 71 valid sites for the analysis having all the above pollutants. The technical details and measurement principle of each of above pollutants can be found in CPCB report [23].

**S2 Background conditions**

Most observational studies [18, 24] draw conclusions based on the anomalies observed during the lockdown period of 2020 compared to those during the same period in 2019. Sicard et al. [25] and Wang [26] discussed the drawbacks of this approach as the variation in the emissions is highly sensitive to prevailing weather conditions that may exhibit variability on a seasonal to inter-annual scale. Therefore, before the gross changes that occurred due to the lockdown can be analyzed, it is important to examine the background meteorological conditions over the study region. Rao and Ramamurti [27], Beegum et al. [28], and Mitra et al. [29] discussed the prevailing weather conditions over India during the months of March and April. The temperatures start to increase over the Deccan plateau and inland regions up to Central India in March, with the highest temperatures observed inland. The gradual increase in temperature over the land, accompanied by the relatively high pressures over the Bay of Bengal (BoB) and the Arabian Sea often lead to large pressure gradients, resulting in the formation of severe convective activities with lighting over central to northern India. Low-level westerly to south-westerly winds, depending on the movement of the low-pressure regions, generally prevail over northern to central India. The strength of westerly winds increases with height and reaches a maximum at approximately 300 hPa as part of the subtropical westerly jet, which oscillates from the north to the south of the peninsula (India) depending on the synoptic conditions [30]. Most of southern India experiences hot and humid conditions, and rare increases in the insolation form low-pressure troughs with associated convective weather systems. The surface winds are mostly easterly to north-easterly, and coastal regions are often dominated by land-sea breeze activity. The boundary layer height (BLH), another influential parameter that plays a critical role in controlling the vertical mixing of pollutants, is relatively high (2–3 km) in the spring as compared to the winter (1–1.5 km) and monsoon (2 km) months, with the maxima located over peninsular India [31, 32].

**References:**

1. Grell G.A., Peckham S.E., R Schmitz, and SA McKeen, G Frost, WC Skamarock, and B Eder. (2005) Fully coupled 'online' chemistry in the WRF model. Atmos. Environ., 39:6957-6976.
2. Gemmill, W., B. Katz, and X. Li (2007) Daily real-time, global sea surface temperature—High-resolution analysis: RTG_SST_HR. Science Application International Corporation and Joint Center for Satellite Data Assimilation, NOAA/NWS/NCEP/EMC/MMAB, Tech. Note 260, 22 pp.
3. Marsh, D., M. J. Mills, D. E. Kinnison, J.-F. Lamarque, N. Calvo, and L. M. Polvani (2013), Climate change from 1850 to 2005 simulated in CESM1(WACCM), J. Clim.,26, 7372–7391, doi:10.1175/JCLI-D-12-00558.
4. Thompson, G., R. M. Rasmussen, and K.Manning, (2004) Explicit forecasts of winter precipitation using an improved bulk microphysics scheme. Part I: Description and sensitivity analysis. Mon. Wea. Rev., 132, 519–542.
5. Hong, S.-Y., and J.-O. J.Lim, (2006) The WRF single-moment 6-class microphysics scheme (WSM6). J. Korean Meteor. Soc., 42, 129–151.
6. Kain, J.S. (2004) The Kain-Fritsch Convective Parameterization: An Update. Journal of Climate and Applied Meteorology, 43, 170-181.
7. Niu, G. Y., Yang, Z. L., Mitchell, K. E., Chen, F., Ek, M. B., Barlage, M., Kumar, A., Manning, K., Niyogi, D., and Rosero, E.: The community Noah land surface model with multiparameterization options (Noah-MP): 1. Model description and evaluation with local-scale measurements, J. Geophys. Res.-Atmos., 116, D12109, https://doi.org/10.1029/2010JD015139, 2011.
8. Iacono, M. J., Delamere, J. S., Mlawer, E. J., Shephard, M. W., Clough, S. A., and Collins, W. D. (2008). Radiative forcing by long‐lived greenhouse gases: Calculations with the AER radiative transfer models, J. Geophys. Res., 113, D13103, doi:10.1029/2008JD009944.
9. Emmons, L. K., Walters, S., Hess, P. G., Lamarque, J.-F., Pfister, G. G., Fillmore, D., Granier, C., Guenther, A., Kinnison, D., Laepple, T., Orlando, J., Tie, X., Tyndall, G., Wiedinmyer, C., Baughcum, S. L., and Kloster, S (2010) Description and evaluation of the Model for Ozone and Related chemical Tracers, version 4 (MOZART-4), Geosci. Model Dev., 3, 43–67, https://doi.org/10.5194/gmd-3-43-2010, 2010.
10. Jones, S. L, Adams-Selin, R., Hunt, E. D., Creighton, G. A., Cetola, J. D. (2012) Update on modifications to WRF-CHEM GOCART for fine-scale dust forecasting at AFWA. https://ui.adsabs.harvard.edu/abs/2012AGUFM.A33D0188J/abstract, AGU Fall Meeting Abstracts.
11. LeGrand, S. L., Polashenski, C., Letcher, T. W., Creighton, G. A., Peckham, S. E., and Cetola, J. D. (2019) The AFWA dust emission scheme for the GOCART aerosol model in WRF-Chem v3.8.1, Geosci. Model Dev., 12, 131–166, <https://doi.org/10.5194/gmd-12-131-2019>.
12. Guenther, A., Karl, T., Harley, P., Wiedinmyer, C., Palmer, P. I., & Geron, C. (2006). Estimates of global terrestrial isoprene emissions using MEGAN (Model of Emissions of Gases and Aerosols from Nature). Atmospheric Chemistry and Physics, 6(11), 3181-3210.
13. Wesely, M. L., & Hicks, B. B. (2000). A review of the current status of knowledge on dry deposition. Atmospheric environment, 34(12-14), 2261-2282.
14. Venkataraman, C., Brauer, M., Tibrewal, K., Sadavarte, P., Ma, Q., Cohen, A., ... & Wang, S. (2018). Source influence on emission pathways and ambient PM 2.5 pollution over India (2015–2050). Atmospheric Chemistry and Physics, 18(11), 8017-8039.
15. Sharma, S., Zhang, M., Anshika, Gao, J., Zhang, H. and Kota, S.H. (2020). Effect of restricted emissions during COVID-19 on air quality in India. Sci. Total Environ. 728: 138878. <https://doi.org/10.1016/j.scitotenv.2020.138878>.
16. Jain, S., and Sharma, T. (2020). Social and travel lockdown impact considering coronavirus disease (COVID-19) on air quality in megacities of India: Present benefits, future challenges and way forward. Aerosol Air Qual. Res. 101343. <https://doi.org/10.1016/j.mvr.2017.09.004>.
17. Ratnam, V., Prasad, P., Raj, S. A., & Ibrahim, H. (2020). Effect of lockdown due to COVID-19 on the aerosol and trace gases spatial distribution over India and adjoining regions. Aerosol and Air Quality Research, 20.
18. Singh, V., Singh, S., Biswal, A., Kesarkar, A. P., Mor, S., & Ravindra, K. (2020). Diurnal and temporal changes in air pollution during COVID-19 strict lockdown over different regions of India. Environmental Pollution, 266, 115368.
19. Kilpatrick, K. A., Podestá, G., Walsh, S., Williams, E., Halliwell, V., Szczodrak, M., Brown, O.B., Minnett, P.J., and Evans, R. (2015). A decade of sea surface temperature from MODIS. Remote Sensing of Environment. 165: 27-41. https://doi.org/10.1016/j.rse.2015.04.023.
20. Jain, C. D., B.L. Madhavan, M. Venkat Ratnam (2019). Source apportionment of rainwater chemical composition to investigate the transport of lower atmospheric pollutants to the UTLS region, Environmental Pollution 248 166e174.
21. Ravi Kiran, S. Talukdar, M. Venkat Ratnam, A. Jayaraman (2018). Long-term observations of black carbon aerosol over a rural location in southern peninsular India: Role of dynamics and meteorology, Atmospheric Environment 189 264–274
22. Corrigan, C. E., Ramanathan, V., and Schauer, J. J. (2006). Impact of monsoon transitions on the physical and optical properties of aerosols, J. Geophys. Res., 111, D18208, doi:10.1029/2005JD006370.
23. CPCB, 2019, Technical Specifications for Continuous Ambient Air Quality Monitoring (CAAQM) Station (2019). https://cpcb.nic.in/report.php.
24. Xu, K., Cui, K., Young, L.H., Hsieh, Y.K., Wang, Y.F., Zhang, J. and Wan, S. (2020). Impact of the COVID-19 event on air quality in central China. Aerosol Air Qual. Res. 20: 915–929. https://doi.org/10.4209/aaqr.2020.04.0150
25. Sicard P, De Marco A, Agathokleous E, et al. (2020) Amplified ozone pollution in cities during the COVID-19 lockdown. Sci Total Environ. 735, 139542. doi:10.1016/j.scitotenv.2020.139542.
26. Wang, P., K. Chen, S. Zhu, P. Wang, H. Zhang. (2020). Severe air pollution events not avoided by reduced anthropogenic activities during COVID-19 outbreak Resour. Conserv. Recycl., 158 (2020), Article 104814
27. Rao, Y. P. and Ramamurthy, K. S. (1968) Climate of India, Forecasting Manual India, Met. Dept., FMU, Rep. 1-2.
28. Beegum, S.N., K.K. Moorthy, S.S. Babu, S.K. Satheesh, V. Vinoj, K.V.S. Badarinath, P.D. Safai, P.C.S. Devara, S. Singh, Dumka U.C. Vinod, P. Pant (2009) Spatial distribution of aerosol black carbon over India during pre-monsoon season Atmos. Environ., 43 (2009), pp. 1071-1078, 10.1016/j.atmosenv.2008.11.042
29. Mitra, C., Shepherd, J.M. and Jordan, T. (2012) On the relationship between the premonsoonal rainfall climatology and urban land cover dynamics in Kolkata city, India. Int. J. Climatol., 32: 1443-1454. doi:10.1002/joc.2366
30. Ramaswamy, C., (1956) On the Sub-tropical Jet Stream and its Role in the Development of Large-scale Convection, Tellus, 8:1, 26-60, DOI: 10.3402/tellusa.v8i1.8943.
31. Patil M.N., Patil S.D., Waghmare R.T., Dharmaraj T. (2013). Planetary Boundary Layer height over the Indian subcontinent during extreme monsoon years. Journal of Atmospheric and Solar Terrestrial Physics, 92, 94-99 DOI:10.1016/j.jastp.2012.10.011.
32. Basha, G., P. Kishore, M. Venkat Ratnam, S. Ravindra Babu, Isabella Velicogna, Jonathan H. Jiang, Chi O. Ao, Global climatology of planetary boundary layer top obtained from multi-satellite GPS RO observations, Climate Dynamics, https://doi.org/10.1007/s00382-018-4269-

**Supplementary Figure captions:**

**Figure S1:** Model domain for simulating the aerosol and trace gases using WRF-Chem. The different regions considered in the present study are also highlighted, including the Gadanki location where ground-based observations are available.

**Figure S2:** Correlation between AERONET AOD and WRF-Chem AOD over three stations during 1 March to 1 May 2020.

**Figure S3**: Spatial correlation between OMI and WRF-Chem outputs in (a) NO_2_ and (b) SO_2_ during 1 March 2020 to 30 April 2020. (c) Spatial correlation between MODIS AOD and WRF-Chem AOD during the same period. Correlations with 95% confidence level are shown with dots.

**Figure S4.** Comparison between AOD as resulting from (a) MODIS measurements and (b) WRF-Chem simulations during 8 March to 20 April 2020 over India and adjoining regions. (c) and (d) same as (a) and (b) but for NO_2_ from OMI and WRF-Chem simulations, respectively. (e) and (f) same as (a) and (b) but for SO_2_ from OMI satellite and WRF-Chem simulations, respectively.

**Figure S5.** Spatial distribution of AOD as infered from MODIS during (a) PLD period, (b) DLD period. (c) Percentage difference in AOD between PLD and DLD periods over India and adjoining regions. (d) to (f) same as (a) to (c) respectively, but simulated by WRF-Chem.

**Figure S6.** Spatial distribution of NO_2_ as infered from OMI during (a) PLD period and (b) DLD period. (c) Percentage difference in NO_2_ between PLD and DLD periods over India and adjoining regions. (d) to (f) same as (a) to (c) but simulated by WRF-Chem.

**Figure S7**. Spatial distribution of BC hydrophobic, BC hydrophilic and their ratio observed during PLD and DLD periods based on WRF-Chem simulations for India and adjacent regions.

**Figure S8.** Percentage difference in the (a) PM_2.5_, (b) PM_10_, (c) BC hydrophobic, (d) BC hydrophilic, (e) OC hydrophobic, and (f) OC hydrophilic observed between 2020 and 2019 during DLD period based on WRF-Chem simulations for India and adjacent regions.

**Figure S9.** Percentage difference in the (a) NO, (b) NO_3_^-^, (c) N_2_O_5_, (d) CO, (e) O_3_, (f) CH_4_, (g) SO_2_, and (h) SO_4_^2-^ observed between the 2020 and 2019 during DLD period based on WRF-Chem simulations for India and adjacent regions.

**Figure S10.** Fire radiative power observed over India and adjoining regions during (a) PLD period, (b) DLD1 period (25 March to 07 April 2020), and (c) DLD2 (8-20 April 2020) as inferred from MODIS observations.

**Figure S11**. Percentage difference in the BLH observed between the PLD and DLD periods based on WRF-Chem simulations for India and the adjacent regions. (b) same as (a) but obtained from ERA-5 reanalysis data sets. (c) Percentage difference in the BLH observed between the DLD periods of 2020 and 2019.

**Figure S12. Mean wind vectors averaged during DLD period at (a) 850 hPa, (b) 700 hPa and (c) 500 hPa different pressure levels as simulated by WRF-Chem.**

**Figure S13. Concentration weighted trajectory (CWT) maps of AOD for Central India during the DLD period. Filled circle (blue color) denote centre of central India.**

**Figure S14.** Percentage change in the wind speed observed at (a) 850 hPa, (b) 700 hPa and (c) 500 hPa between PLD and DLD periods over India and adjoining regions. (d) to (e) same as (a) to (c) but for RH.

**Supplementary Figures:**


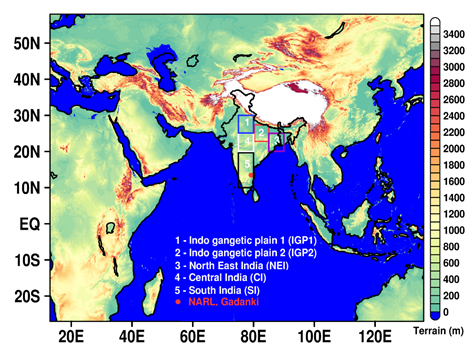


**Figure S1:** Model domain used for simulating aerosol and trace gases using WRF-Chem. The different regions considered in the present study are also highlighted, including Gadanki, for which ground-based observations are available. The figure is plotted using freely available software GrADS (Grid Analysis and Display system).


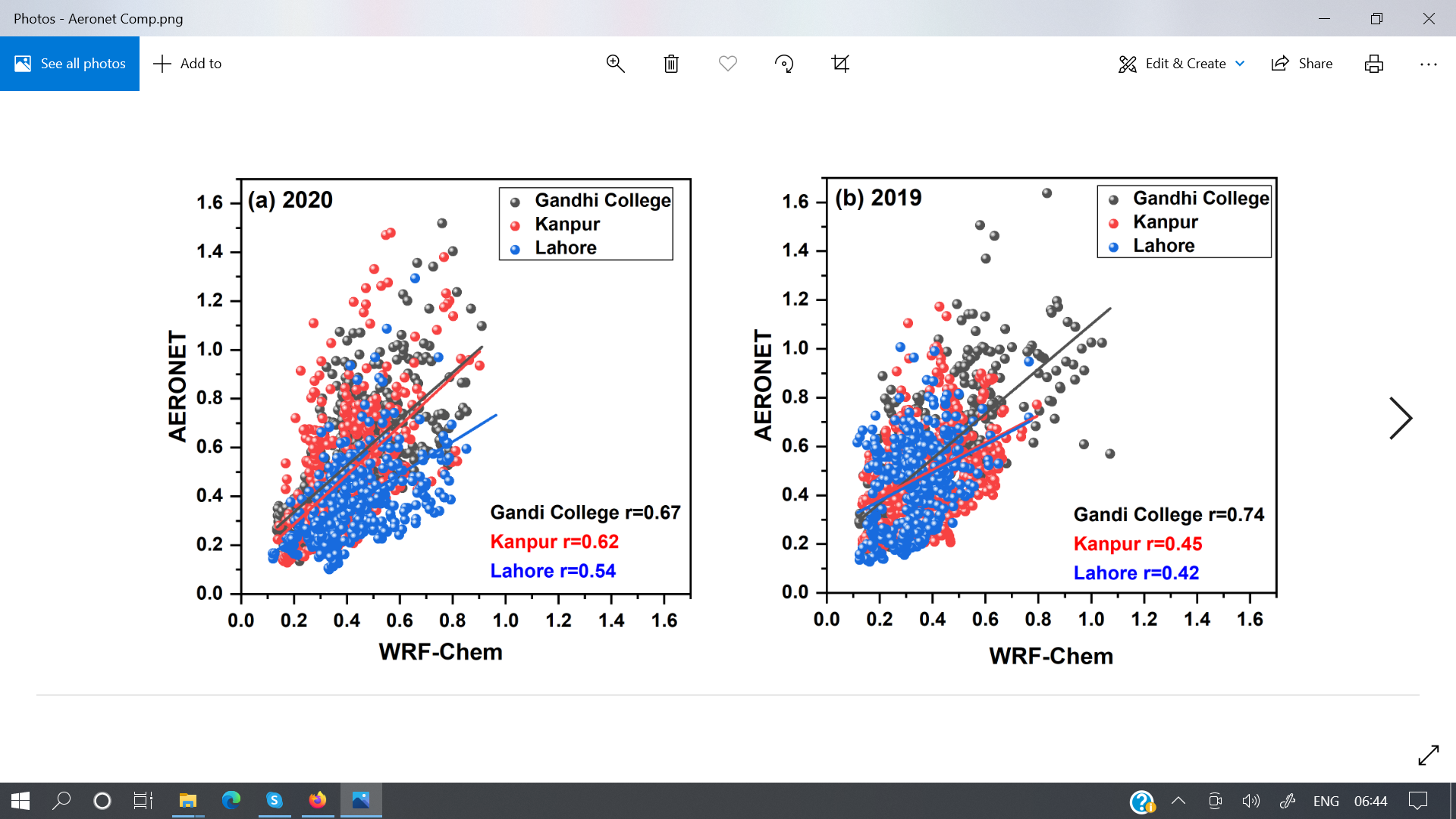


**Figure S2:** Correlation between AERONET AOD and WRF-Chem AOD over three stations during 1 March to 1 May 2020. The figure is plotted using ORIGIN software (https://www.originlab.com).


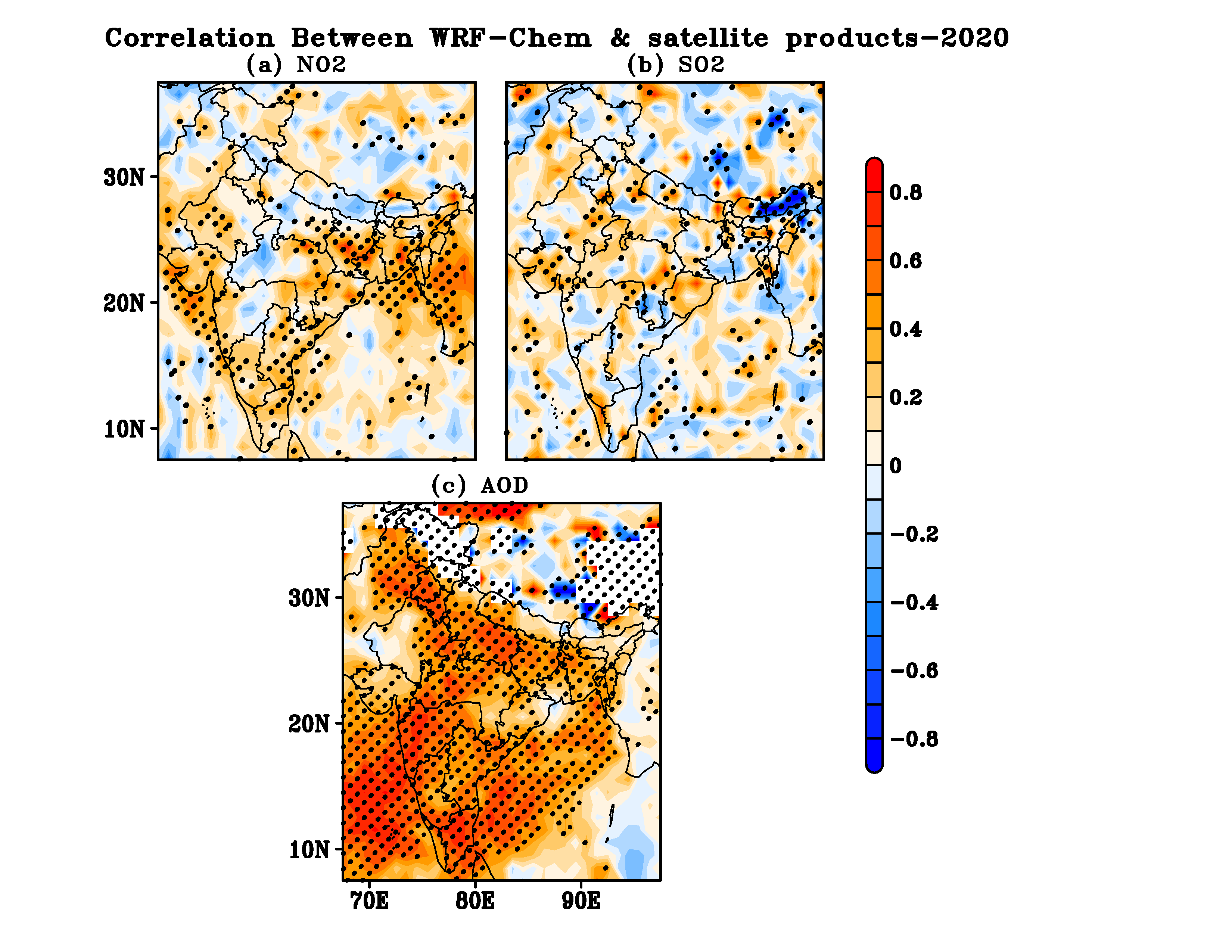


**Figure S3**: Spatial correlation between OMI and WRF-Chem outputs in (a) NO_2_ and (b) SO_2_ during 1 March 2020 to 30 April 2020. (c) Spatial correlation between MODIS AOD and WRF-Chem AOD during the same period. Correlations with 95% confidence level are shown with dots. The figures are plotted using GrADS V2.2.1 software (http://cola.gmu.edu/grads/).


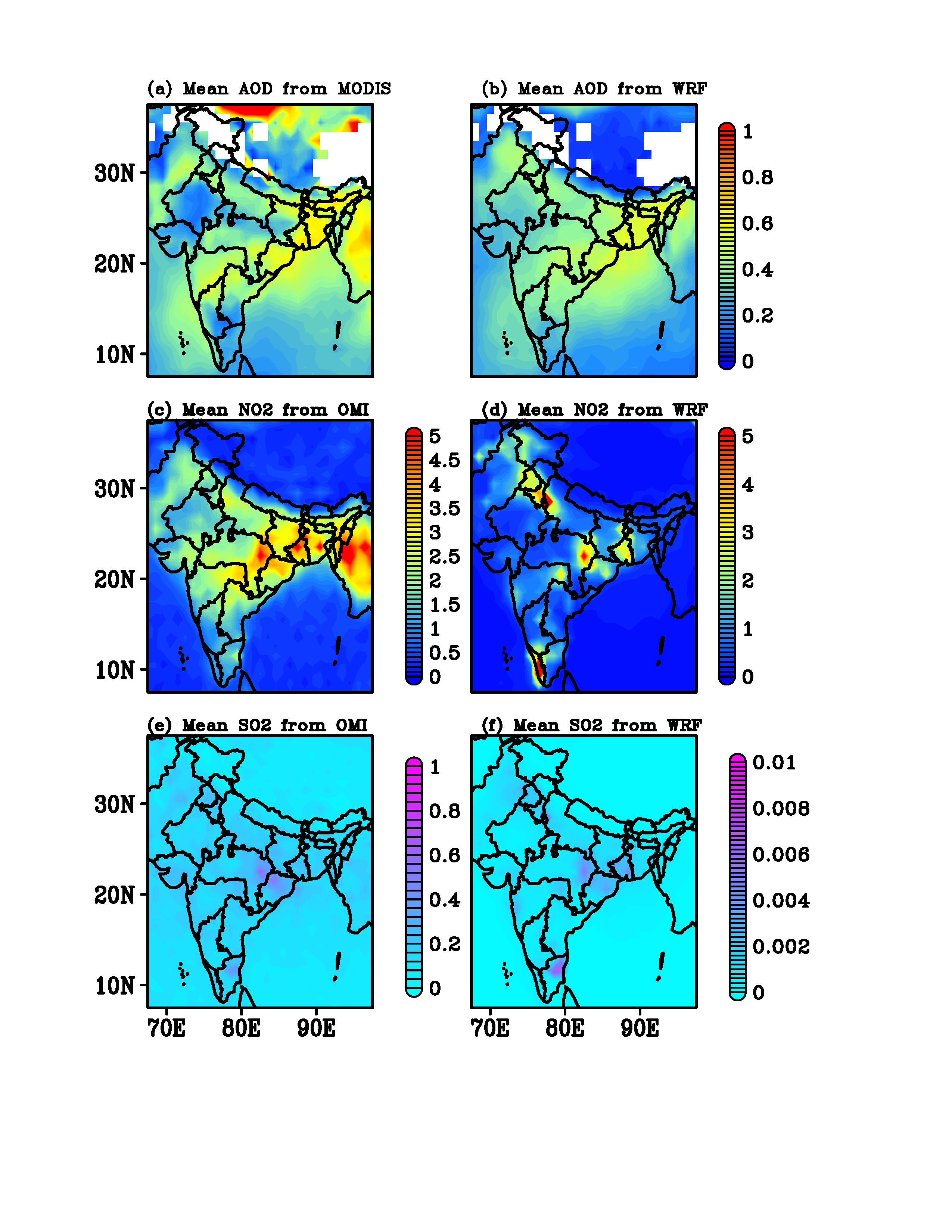


**Figure S4.** Comparison between the AOD resulting from (a) MODIS measurements and (b) WRF-Chem simulations during the period March 8 to April 20, 2020 for India and the adjacent regions. (c) and (d) are the same as (a) and (b) but for NO_2_ from OMI and WRF-Chem simulations, respectively. (e) and (f) are the same as (a) and (b) but for SO_2_ from OMI satellite and WRF-Chem simulations, respectively. The figures are plotted using GrADS V2.2.1 software (http://cola.gmu.edu/grads/).


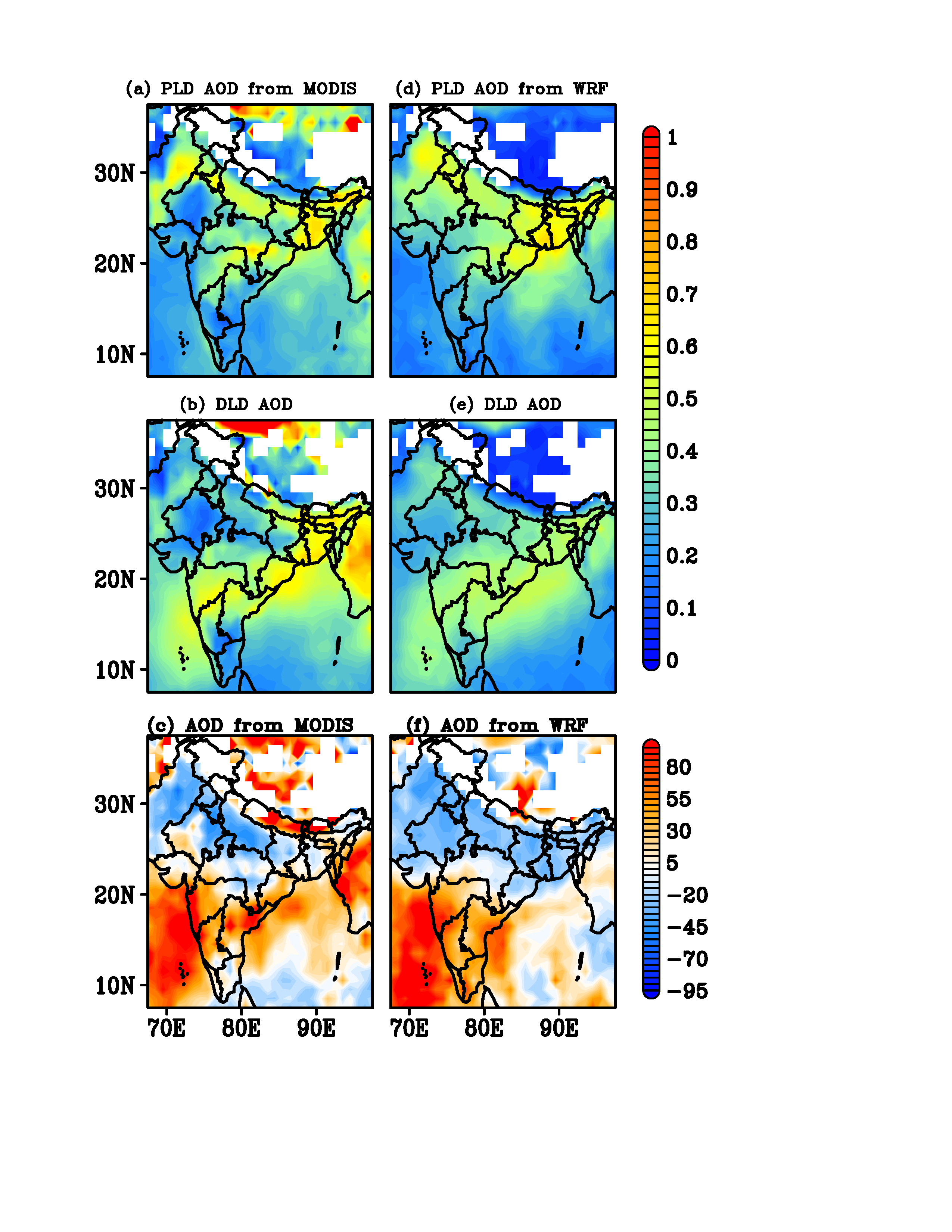


**Figure S5.** Spatial distribution of AOD as inferred using data from MODIS during (a) PLD period, (b) DLD period. (c) Percentage difference in AOD between PLD and DLD periods over India and the adjacent regions. (d) to (f) are the same as (a) to (c), respectively, but simulated by WRF-Chem. The figures are plotted using GrADS V2.2.1 software (http://cola.gmu.edu/grads/).

**
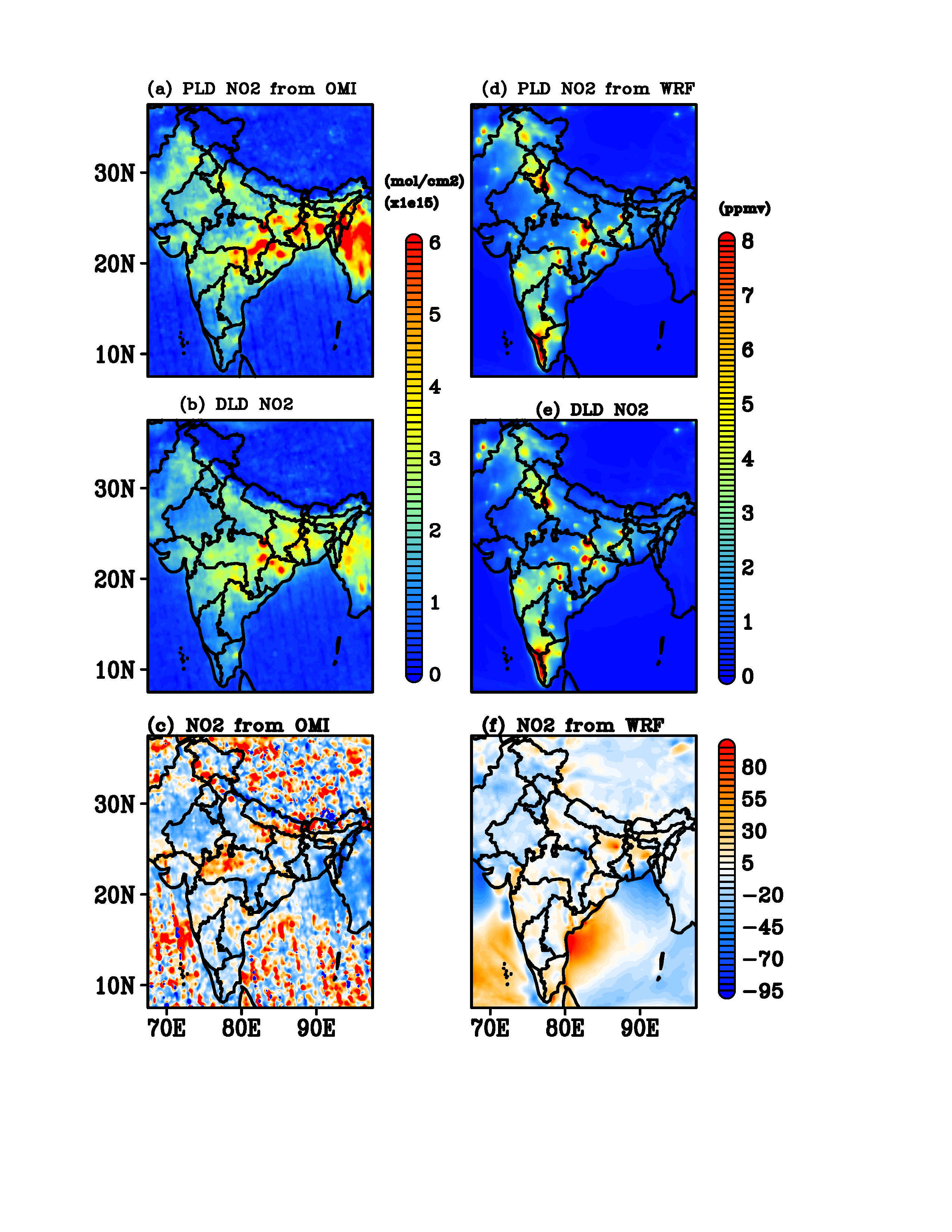
**

**Figure S6.** Spatial distribution of NO_2_ as infered from OMI during (a) PLD period and (b) DLD period. (c) Percentage difference in NO_2_ between PLD and DLD periods over India and the adjacent regions. (d) to (f) are the same as (a) to (c) but simulated by WRF-Chem. The figures are plotted using GrADS V2.2.1 software (http://cola.gmu.edu/grads/).


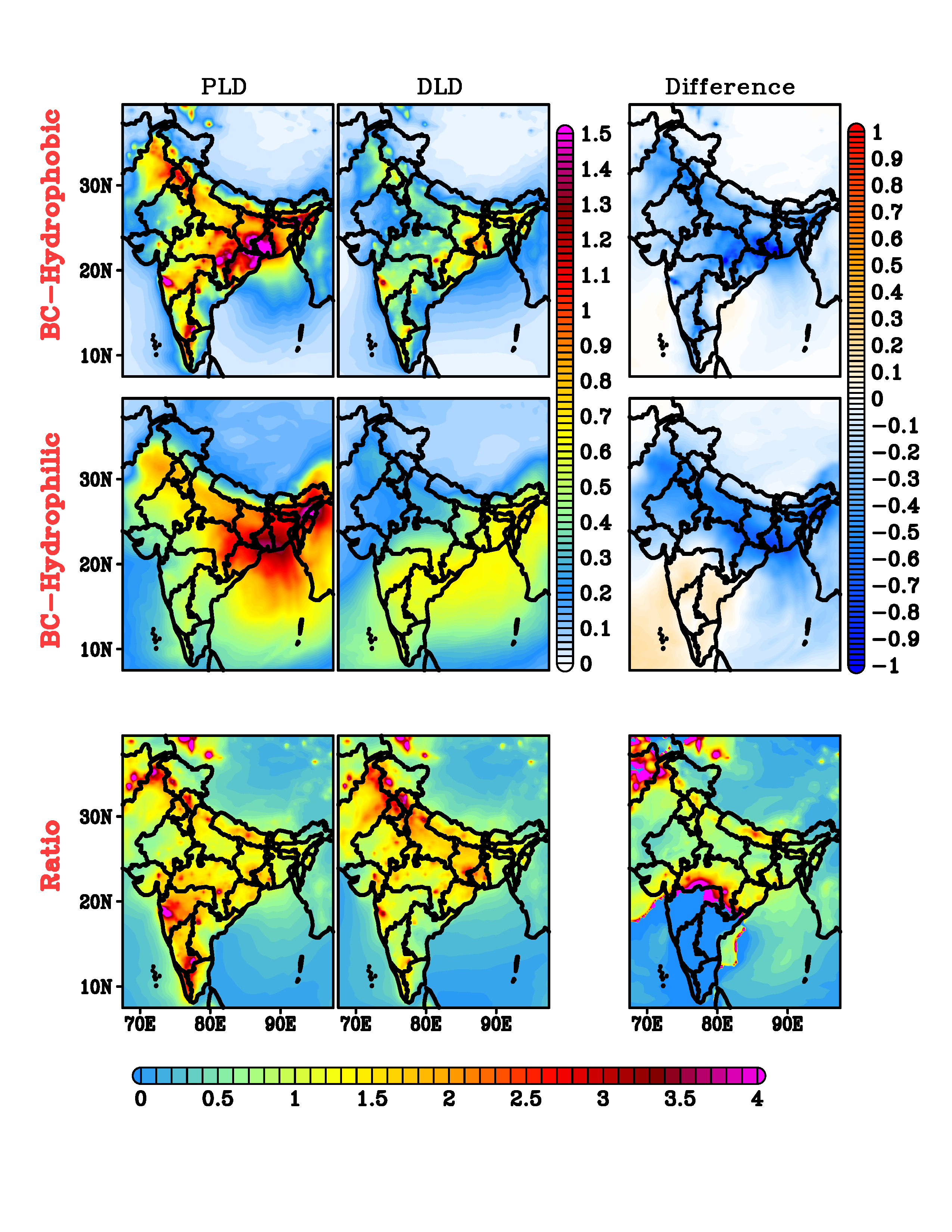


**Figure S7**. Spatial distribution of BC hydrophobic, BC hydrophilic and their ratio observed during PLD and DLD periods based on WRF-Chem simulations for India and adjacent regions. The figures are plotted using GrADS V2.2.1 software (http://cola.gmu.edu/grads/).


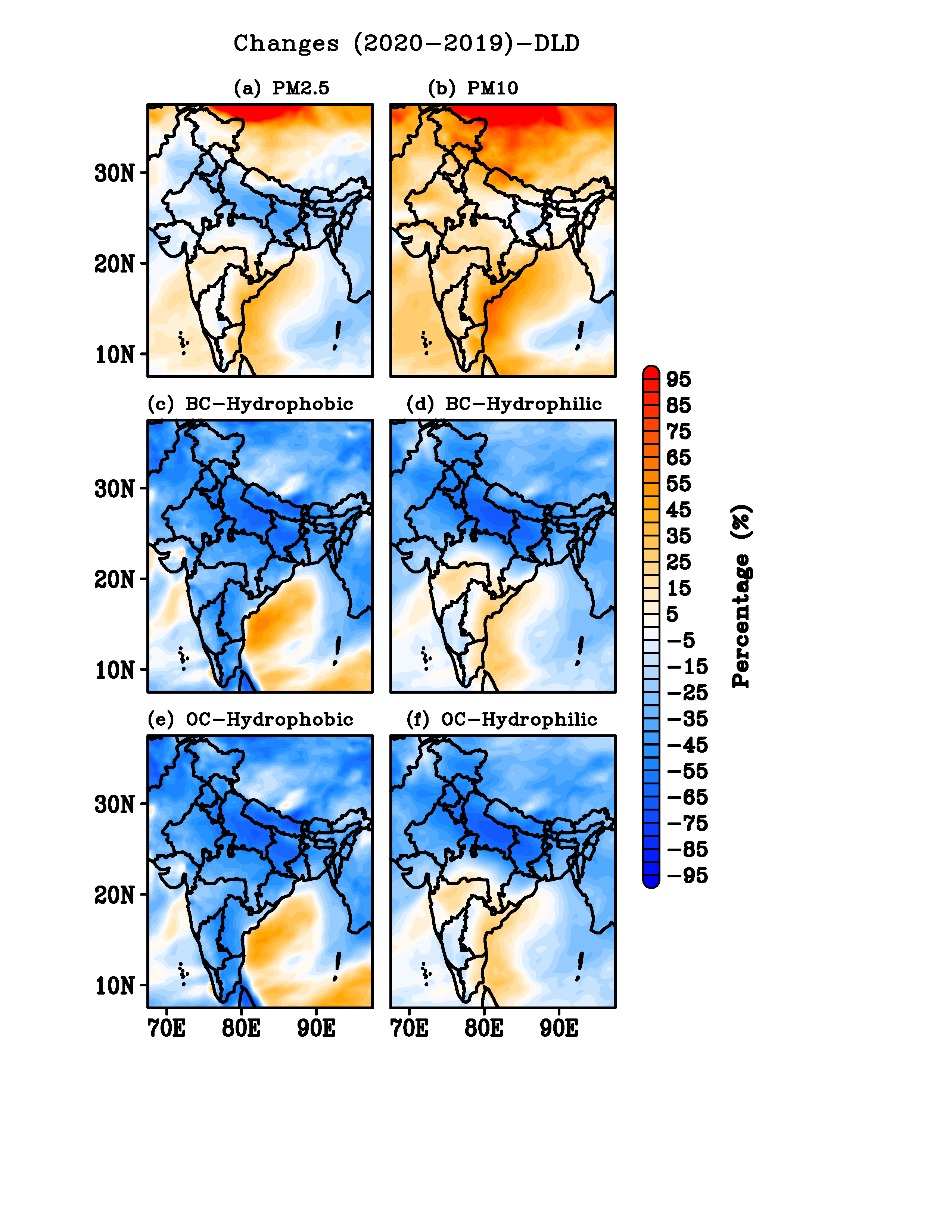


**Figure S8.** Percentage difference in the (a) PM_2.5_, (b) PM_10_, (c) BC hydrophobic, (d) BC hydrophilic, (e) OC hydrophobic, and (f) OC hydrophilic observed between 2020 and 2019 during DLD period based on WRF-Chem simulations for India and adjacent regions. The figures are plotted using GrADS V2.2.1 software (http://cola.gmu.edu/grads/).


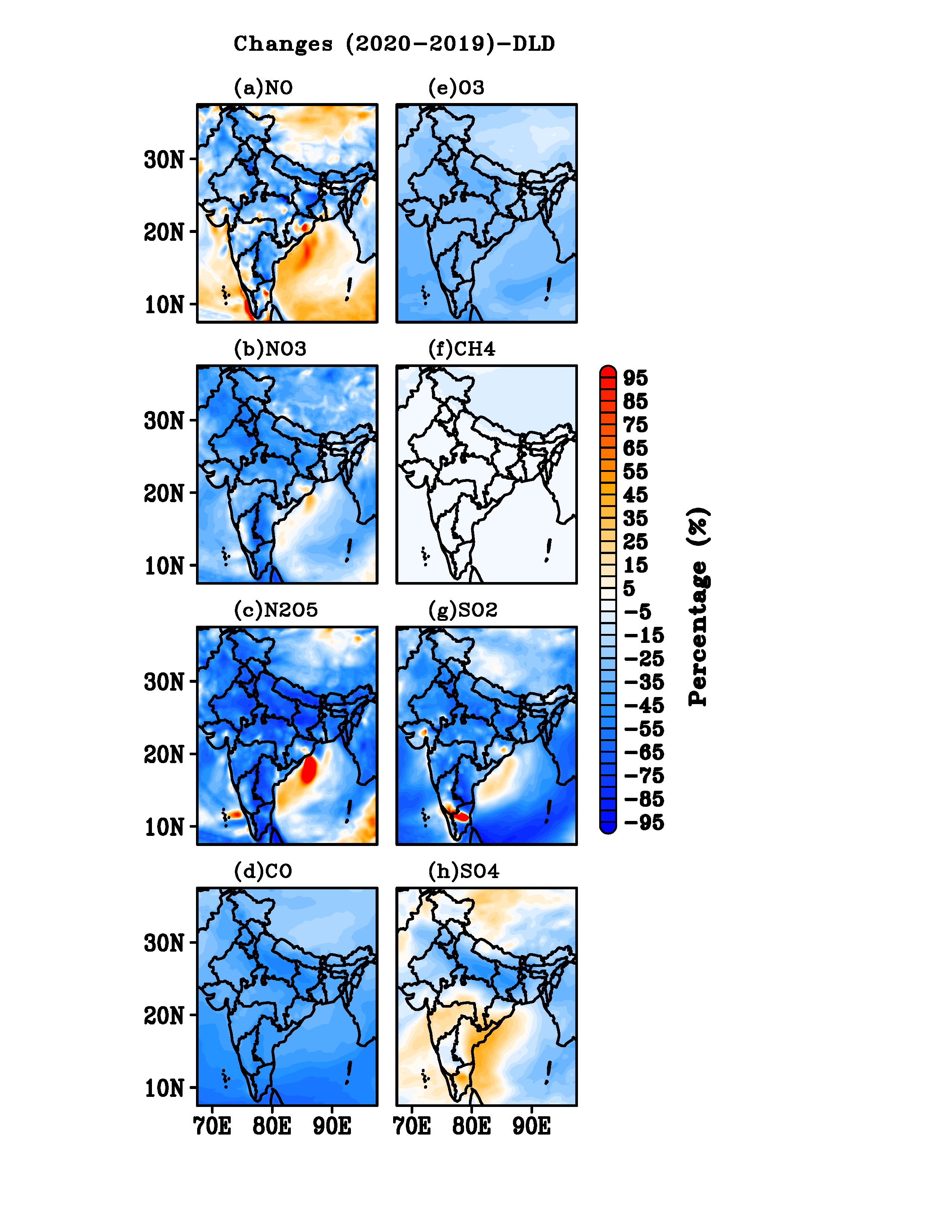


**Figure S9.** Percentage difference in the (a) NO, (b) NO_3_^-^, (c) N_2_O_5_, (d) CO, (e) O_3_, (f) CH_4_, (g) SO_2_, and (h) SO_4_^2-^ observed between the 2020 and 2019 during DLD period based on WRF-Chem simulations for India and adjacent regions. The figures are plotted using GrADS V2.2.1 software (http://cola.gmu.edu/grads/).


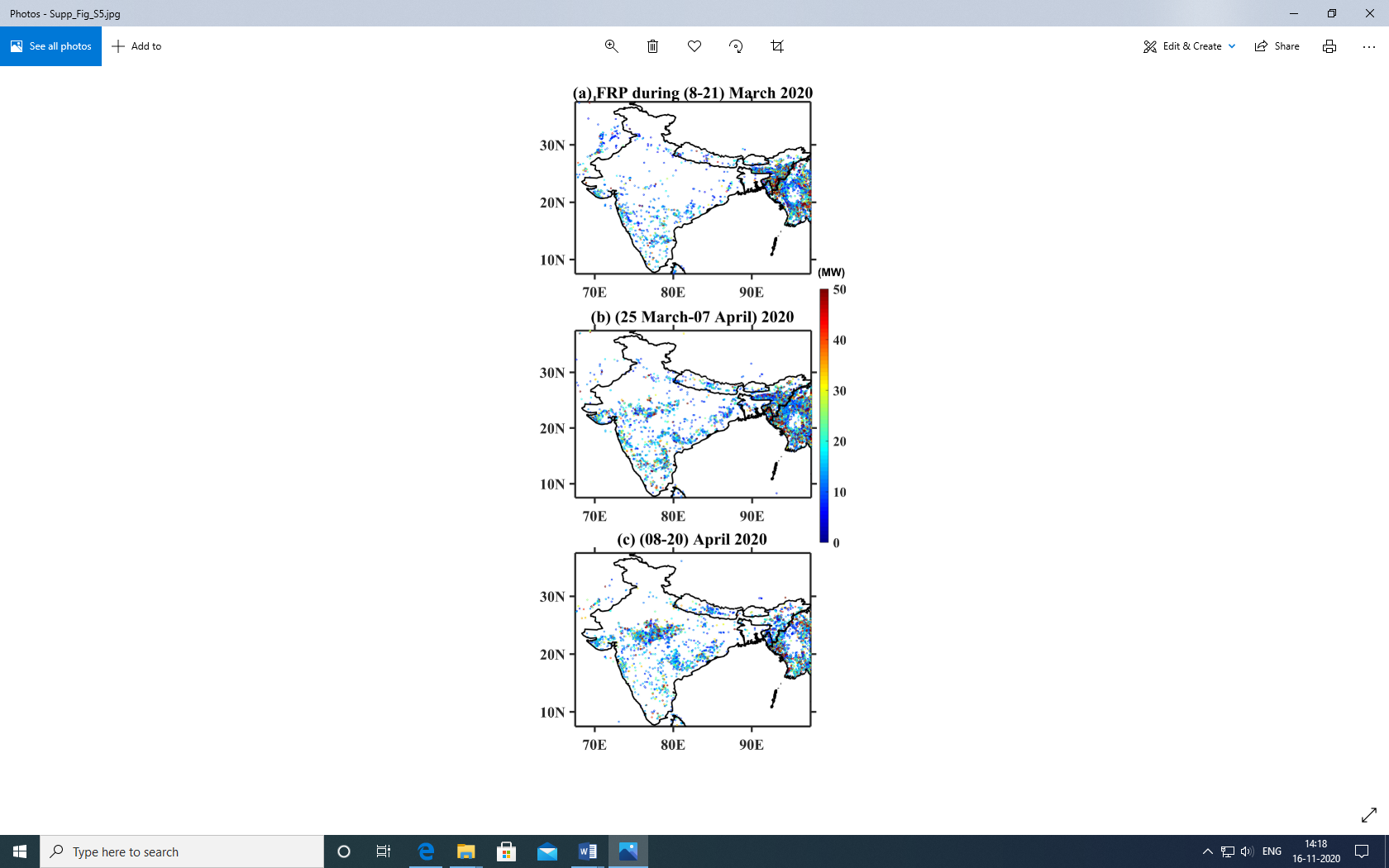


**Figure S10.** The FRP observed over India and the adjacent regions during (a) PLD period, (b) DLD1 period (March 25 to April 7, 2020), and (c) DLD2 (April 8–20, 2020) as inferred from MODIS observations. The figures are plotted using GrADS V2.2.1 software (http://cola.gmu.edu/grads/).


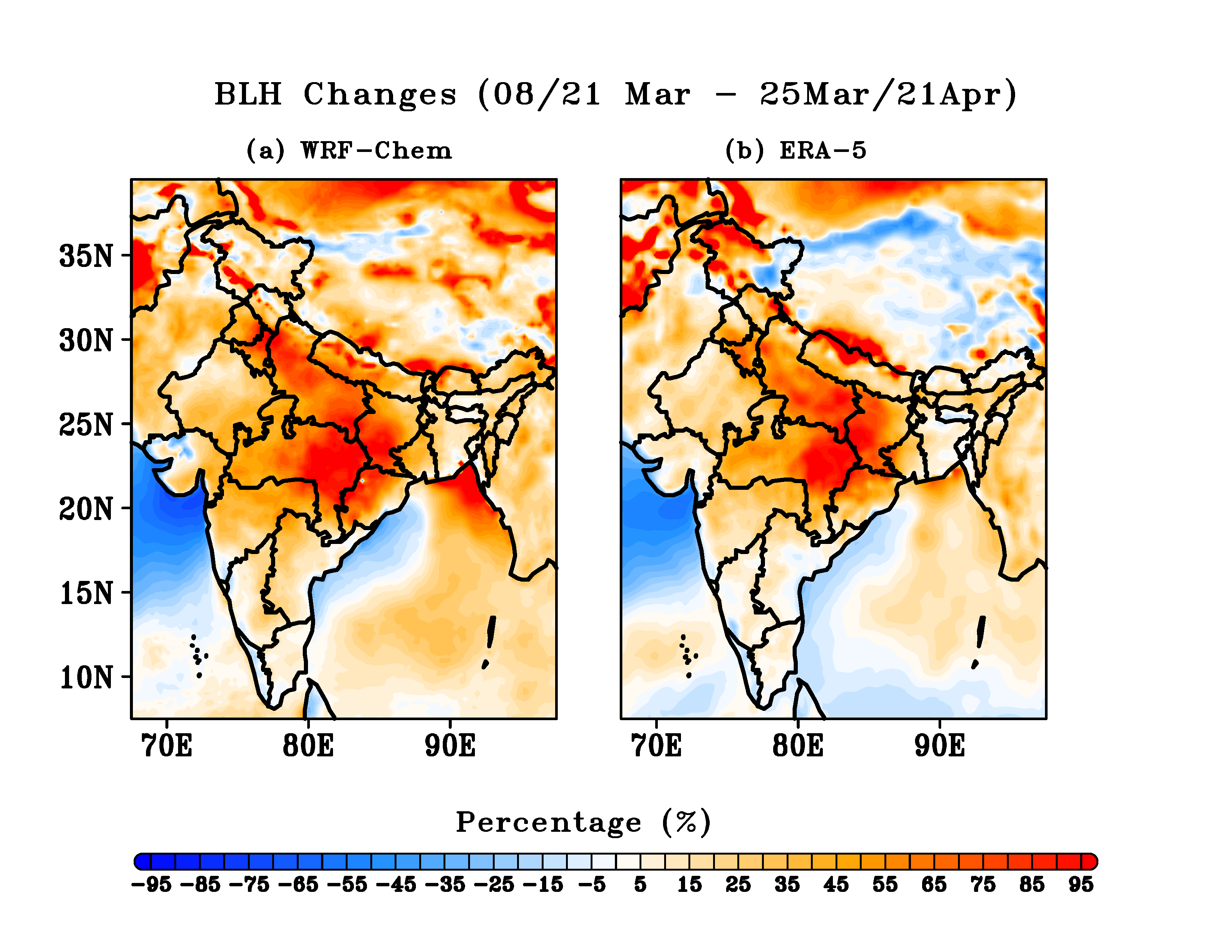

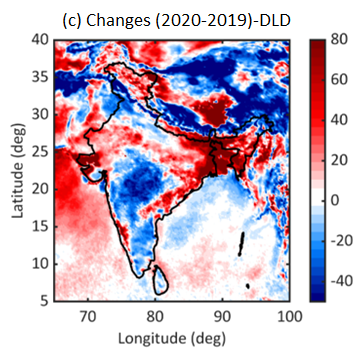


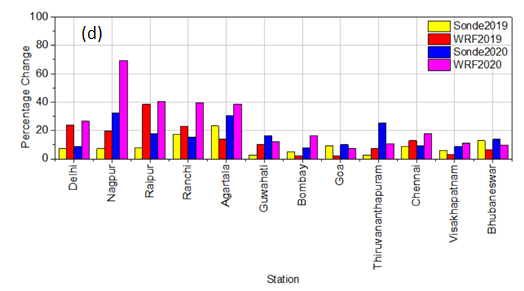


**Figure S11**. Percentage difference in the BLH observed between the PLD and DLD periods based on WRF-Chem simulations for India and the adjacent regions. (b) same as (a) but obtained from ERA-5 reanalysis data sets. (c) Percentage difference in the BLH observed between the DLD periods of 2020 and 2019. (d) Percentage change in BLH obtained from network of radiosondes during 2019 and 2020 and its comparison with WRF-Chem. The figures are plotted using GrADS V2.2.1 software (http://cola.gmu.edu/grads/).


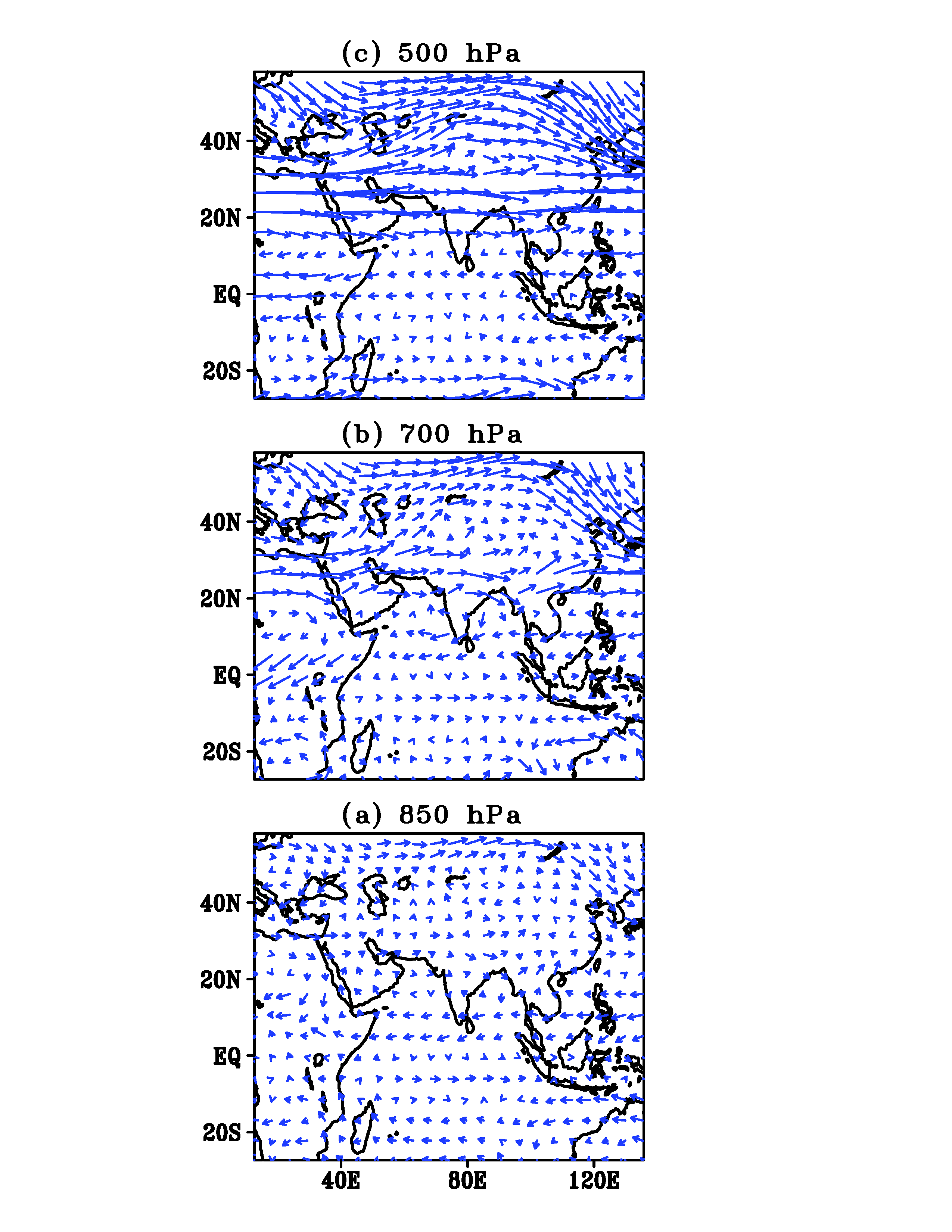


**Figure S12.** Mean wind vectors averaged from WRF-Chem simulations during the DLD period at different pressure levels: (a) 850 hPa, (b) 700 hPa, and (c) 500 hPa. The figures are plotted using GrADS V2.2.1 software (http://cola.gmu.edu/grads/).


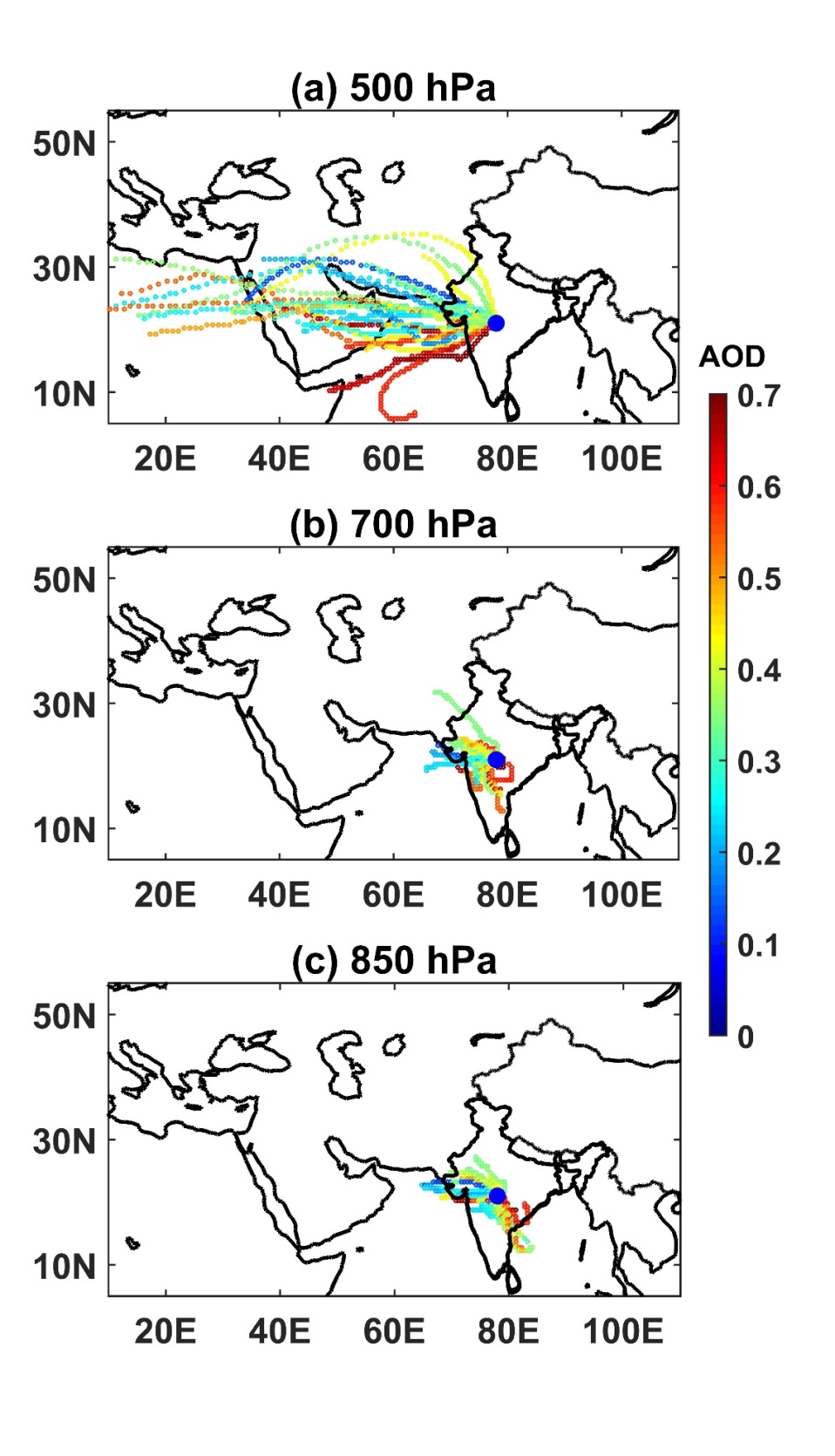


**Figure S13. Concentration weighted trajectory (CWT) maps of the AOD for Central India during the DLD period. Filled circle (blue) denotes the center of central India.** The figures are plotted using GrADS V2.2.1 software (http://cola.gmu.edu/grads/).


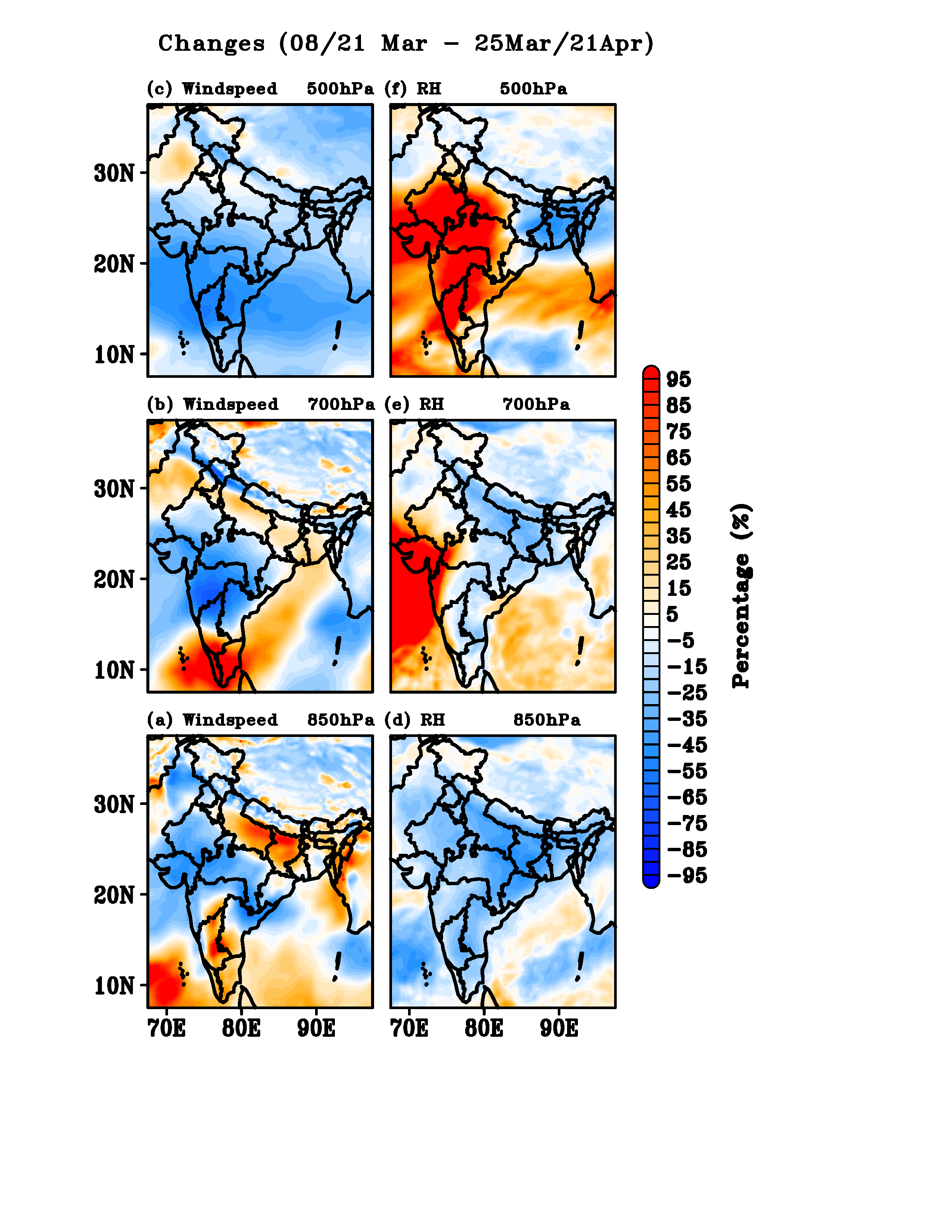


**Figure S14.** Percentage change in the wind speed observed at (a) 850 hPa, (b) 700 hPa, and (c) 500 hPa between the PLD and DLD periods in India and adjacent regions. (d) to (f) are the same as (a) to (c) but for Relative Humidity. The figures are plotted using GrADS V2.2.1 software (http://cola.gmu.edu/grads/).
